# IκBα targeting promotes oxidative stress-dependent cell death

**DOI:** 10.1186/s13046-021-01921-x

**Published:** 2021-04-16

**Authors:** Giovanna Carrà, Giuseppe Ermondi, Chiara Riganti, Luisella Righi, Giulia Caron, Alessio Menga, Enrica Capelletto, Beatrice Maffeo, Marcello Francesco Lingua, Federica Fusella, Marco Volante, Riccardo Taulli, Angelo Guerrasio, Silvia Novello, Mara Brancaccio, Rocco Piazza, Alessandro Morotti

**Affiliations:** 1 Department of Clinical and Biological Sciences, University of Turin, Regione Gonzole 10, 10043 Orbassano, Italy; 2 Department of Molecular Biotechnology and Health Sciences, University of Turin, Via Nizza 52, 10126 Turin, Italy; 3 Department of Oncology, University of Turin, Regione Gonzole 10, 10043 Orbassano, Italy; 4 Department of Medical Science, University of Turin, Turin, Italy; 5 Department of Medicine and Surgery, University of Milano-Bicocca and San Gerardo Hospital, 20900 Monza, Italy

**Keywords:** NFKBIA, OXPHOS, Oxidative stress, Programmed cell death, Oxytosis, IκBα

## Abstract

**Background:**

Oxidative stress is a hallmark of many cancers. The increment in reactive oxygen species (ROS), resulting from an increased mitochondrial respiration, is the major cause of oxidative stress. Cell fate is known to be intricately linked to the amount of ROS produced. The direct generation of ROS is also one of the mechanisms exploited by common anticancer therapies, such as chemotherapy.

**Methods:**

We assessed the role of NFKBIA with various approaches, including in silico analyses, RNA-silencing and xenotransplantation. Western blot analyses, immunohistochemistry and RT-qPCR were used to detect the expression of specific proteins and genes. Immunoprecipitation and pull-down experiments were used to evaluate protein-protein interactions.

**Results:**

Here, by using an in silico approach, following the identification of NFKBIA (the gene encoding IκBα) amplification in various cancers, we described an inverse correlation between IκBα, oxidative metabolism, and ROS production in lung cancer. Furthermore, we showed that novel IκBα targeting compounds combined with cisplatin treatment promote an increase in ROS beyond the tolerated threshold, thus causing death by oxytosis.

**Conclusions:**

NFKBIA amplification and IκBα overexpression identify a unique cancer subtype associated with specific expression profile and metabolic signatures. Through p65-NFKB regulation, IκBα overexpression favors metabolic rewiring of cancer cells and distinct susceptibility to cisplatin. Lastly, we have developed a novel approach to disrupt IκBα/p65 interaction, restoring p65-mediated apoptotic responses to cisplatin due to mitochondria deregulation and ROS-production.

**Supplementary Information:**

The online version contains supplementary material available at 10.1186/s13046-021-01921-x.

## Background

Worldwide, non-small cell lung cancer (NSCLC) is the most common cause of cancer death, with an estimated 1.6 million deaths per year [1]. Despite the increased knowledge of the disease over the past two decades, and the impressive results achieved with immunotherapy, the overall survival rate remains low. Platinum-based doublet therapy is the standard therapy for those patients presenting with advanced stage solid cancers at diagnosis. Unfortunately, chemotherapy refractoriness and/or the insensitivity to conventional drugs remain a challenging issue and an urgent clinical need to be solved [2]. Reactive Oxygen Species (ROS) are highly reactive oxygen-containing molecules resulting from cellular hypermetabolism [3]. ROS production is linked to oxidative phosphorylation (OXPHOS) hyperactivity and anti-oxidant machinery impairment and is known to induce per se mitochondria dysfunction [4]. If not eliminated, ROS increase the oxidative damage to mitochondrial proteins and feed their own further production as in a vicious circle [5], which ultimately lead to the apoptosis induction [6]. Moreover, recent studies have provided preclinical evidence that ROS drives the cellular response to DNA damage caused by genotoxic agents such as chemo- and radiotherapy. As a consequence, the development of agents able to modulate ROS production in combination with chemotherapy and radiotherapy represent a challenging therapeutic strategy [7, 8]. The aim of pharmacological approaches based on ROS production is to overcome the threshold that makes cancers cells more vulnerable and prone to death than normal cells [9]. One of the signaling pathways capable of modulating cellular metabolism, ROS production and apoptosis sensitivity is the NFκB pathway [10–13]. Here, we unravel a novel NFKBIA amplification in lung cancer and demonstrate a strong link between IκBα overexpression, low-ROS levels and chemoresistance.

## Methods

### Cohorts of patients and cell lines

Main clinical features of lung cancer patients from San Luigi Gonzaga Hospital (a total 57 samples used for IHC) are summarized in Table S3. Analysis of tumor samples was performed following informed consent and with obscured identity (#232/INT). Human lung cancer cell lines A549, H1299, H460 and H522, as well as non-cancer lines HEK 293 T and BEAS (a kind gift from Prof. R. Taulli and Prof.sa M. Brancaccio) were cultured in cultured in RPMI 1640 medium supplemented with 10% FBS 1% glutamine and 1% penicillin/streptomycin. All cell lines were maintained in a humidified incubator with 5% CO_2_ at 37 °C. Each cell line identity was verified by Short Tandem Repeat (STR) DNA profiling.

### Plasmids and shRNA

The following shRNAs were obtained from Open Biosystems: GIPZ Non-silencing Lentiviral shRNA Control, GIPZ Human NFKBIA shRNA D2 and shRNA D7. The following shRNA was obtained from Sigma: human PLKO.1 pure derived vector RelA. The following plasmid were obtained from Addgene: RelA cFlag pcDNA3 plasmid, pCMV-VSV-G, pCMV-dR8.2 dvpr, p1242-3x-KB-L. IκBα was cloned into myc-tagged pRK5 plasmid. The following plasmid were obtained from Promega: pRL-TK Vector and pGL3 Luciferase Reporter Vectors.

### Antibodies and reagents

Commercially antibodies were used: Vinculin (Sigma, SAB4200080, 1:8000), IκBα (Cell Signaling, #9242, 1:1000), P65 (Cell Signaling, #8242, 1:1000), FLAG (abcam, ab1162), 1:1000), Myc (Cell Signaling, #2276, 1:1000), MITOFUSIN1 (Cell Signaling, #14739, 1:1000), OPA1 (Cell Signaling, #67589, 1:1000), Total OXPHOS Rodent WB Antibody Cocktail antibodies (Abcam, ab110413, 1:1000), peroxidase-conjugated secondary mouse (Cell Signaling, #7076, 1:2000) and peroxidase-conjugated secondary Rabbit (Cell Signaling, #7074, 1:2000). TNFα (300-01A and 315-01A) was purchased from Peprotech. Cisplatin (S1166) was purchased from Selleckchem. ZINC639309 (MolPort-002-836-946) was purchased from MolPort and Psammaplin-A (SC-258049) was purchased from Santa Cruz. NAC (N-acetylcysteine) was purchased from Sigma Aldrich.

### Cell proliferation assay and assessment of apoptosis

For proliferation assay, cells were plated in 96-well plates at a density of 2 × 10^3^ per well. Proliferation was evaluated by CellTiter-Glo. Apoptosis was measured by flow cytometry after staining with Annexin V. Cells were analyzed by FACScelesta using CellQuest Software.

### Lentiviral transduction

Virus containing supernatants were collected 48 h after co-transfection of pCMV-VSV-G, pCMV-dR8.2 dvpr and the shRNA vector into HEK293T cells, and then added to the target cells. Cells were then selected with 10 μg ml^− 1^ puromycin.

### Cell lysis, Western blot analysis and Immunoprecipitation

Lung cancer cells lines were lysed with a total lysis buffer (NaCl 150 mM, EDTA 1 mM, Hepes pH 7.5 50 mM, Triton X 1%, Glycerol 10%) supplemented with protease and phosphatase inhibitors cocktail. After the lysis, cells were centrifuged at 14000 rpm for 15 min. Protein concentration was evaluated using Bradford Reagent, 5x concentrate assay. Total protein extracts (30–50 μg) were boiled into Laemmli Sample buffer (2X) for 5 min and analyzed by Western blotting, using a 4–15% Mini-PROTEAN TGX Stain-Free precast gels. After electrophoresis, the proteins were transferred to a 0.45 nitrocellulose filters (GE Healthcare, Life Sciences #10600003). Immunoblots were then probed overnight at 4 °C with specific antibodies in DPBS-0,1% tween-1% BSA and proteins detection was performed by using appropriate peroxidase-conjugated secondary antibodies and chemiluminescence reagent (BIORAD, #170–5060). Immunoprecipitation was performed using whole-cell lysates. Proteins were extracted with lysis buffer containing 150 mM NaCl, 1 mM EDTA, 50 mM Hepes (pH 7.5), 1% Triton X-100 and 10% glycerol. Then lysates were incubated with antibodies overnight at 4 °C on a rotator. Protein A/G-PLUS-Agarose beads (Santa Cruz, #2003) were added and incubated for 2 h at 4 °C with rotation. Beads bound with immunoreactive complexes were washed four times with cold lysis buffer. Immunoprecipitated complexes were boiled for 5 min and subsequently analyzed with Western Blot.

### Pull-down experiments

For pull-down experiments glutathione-coupled Sepharose 4B beads bound to recombinant GST-PAK CRIB (GE Healthcare, Life Sciences #606-60A) domain fusion proteins were incubated with cell extracts at 4 °C for 1 h, eluted in Laemmli Sample buffer and analyzed with Western Blot for the presence of IκBα.

### Sensor vector generation and reporter assays

For NF-κB Luciferase Assay we used p1242-3x-KB-L containing 3 NF-kappaB binding sites upstream of the Firefly Luciferase gene. For the Luciferase Assay 3 × 10^5^ A549 cells previously described were plated on a 12-well plate. After 24 h, cells were co-transfected using Lipofectamine 2000 with 150 ng of pRL-TK Vector containing the Renilla luciferase construct, used as a normalizer and internal control, and with 650 ng of reporter vector (p1242-3x-KB-L), or with empty vector pGL3 Luciferase Reporter Vectors. After 24 h transfection 24 h Dual-Luciferase Reporter Assay was performed by Glomax instrument. Results are calculated as fold changes and shown as means of Firefly Luciferase activity normalized on Renilla luciferase activity or on total protein extraction.

### Mitotracker

For Mitotracker (Invitorgen, #M7510) cells were plated at confluence 2.5 × 10^5^ in 6 well. Mitotracker was added to the culture medium to a final concentration of 10 nM for 15 min and left at 37 °C. Cells were analyzed by FACScelesta using CellQuest Software (BD Biosciences) or evaluated by Immunofluorescence.

### Ros production

ROS production was followed using MitoSOX (Invitrogen, #M36008) or DCFDA / H2DCFDA - Cellular ROS Assay Kit (ab113851). Cells were incubated for 10 min at 37 °C with 5 μM Mitosox or 20 μM DCFDA / H2DCFD and were washed once. After incubation, fluorescence was measured by FACScelesta using CellQuest Software (BD Biosciences).

### Mito stress test

OCR measurements were conducted using a Seahorse XFe96 analyzer according to manufacturer’s protocol. One day prior to performing the assay, A549 and H460 cells were seeded at 35,000 per well in XFe96 cell culture plates, treated with PSA and incubated in 5% CO2 at 37 °C. One hour prior to analysis, growth medium was replaced with assay medium (DMEM minus phenol red and sodium bicarbonate (Corning, Cat. No. 90–013-PB) that is supplemented with 1 mM pyruvate, 2 mM l-glutamine, and 10 mM glucose, pH 7.4) and incubated in a non-CO2 incubator. During assay, 1 μM oligomycin (Sigma, Cat. No. 495455), 2 μM FCCP (Sigma, Cat. No. C2920), and 1 μM rotenone/antimycin A (Sigma Cat. No. R8875 and A8674) were sequentially injected into each well in accordance with standard protocols. Absolute rates (p moles/min) were normalized to μg of protein.

### Lactate measurement

The release of lactate in cell culture medium was measured using the Lactate Assay kit (Sigma Aldrich #MAK065) as per manufacturer’s instructions. Results were expressed as ng lactate/μl.

### Electron transport chain measurement

To isolate mitochondria, cells were washed twice in ice-cold PBS, lysed in 0.5 ml mitochondria lysis buffer (50 mM Tris, 100 mM KCl, 5 mM MgCl2, 1.8 mM ATP, 1 mM EDTA, pH 7.2), supplemented with protease inhibitor cocktail III (Calbiochem, La Jolla, CA, USA, #539134), 1 mmol/l PMSF and 250 mM NaF. The samples were clarified by centrifugation at 650 g for 3 min at + 4 °C: the supernatant was collected and centrifuged at 13000 g for 5 min at + 4 °C. The supernatant, corresponding to the cytosolic fraction, was transferred into a new series of tubes. The pellet, containing mitochondria, was washed once with lysis buffer, and resuspended in 0.25 ml of a resuspension buffer composed of 250 mM sucrose, 15 mM K2HPO4, 2 mM MgCl2, 0.5 mM EDTA. A 50 μl aliquot was sonicated and used for the measurement of protein content or western blotting. To confirm the presence of mitochondrial proteins in the extracts, 10 μg of each sonicated sample were subjected to SDS-PAGE and probed with an anti-porin antibody (Abcam, Cambridge, UK). To measure the electron flux from complex I to complex III, taken as index of the mitochondrial respiratory activity, 50 μg of proteins, derived from non-sonicated mitochondrial samples, were re-suspended in 0.2 ml of buffer A (5 mM KH2PO4, 5 mM MgCl2, 5% w/v BSA) and transferred into a 96-well plates. Then 0.1 ml of buffer B (25% w/v saponin, 50 mM KH2PO4, 5 mM MgCl2, 5% w/v BSA, 0.12 mM cytochrome c-oxidized form, 0.2 mM NaN3) was added for 5 min at room temperature. The reaction was started with 0.15 mM NADH and was followed for 5 min, reading the absorbance at 550 nm by a Packard microplate reader EL340 (Bio-Tek Instruments, Winooski, VT, USA). The results were expressed as nmoles of cytochrome c reduced/min/mg mitochondrial protein.

### Cholesterol esters measurement and Lipoperoxidation measurement

The amount of cholesterol esters was measured spectrofluorimetrically using the Cholesterol/ Cholesterol Ester Assay Kit–Quantitation Kit (Abcam), following the producer’s instructions. The amount of cholesterol esters was obtained by subtracting the value of free cholesterol from the value of total cholesterol. Results were expressed as μmoles cholesterol esters/mg cell proteins. The amount of Thiobarbituric Acid Reactive Substances (TBARS), an index of lipid peroxidation, was quantified spectrophotometrically with the TBARS Assay Kit (Cayman Chemicals). Results were expressed as nmoles/mg.

### Fatty acids β-oxidation

Cells were washed twice with PBS, detached with trypsin/EDTA (0.05/0.02% v/v) and centrifuged at 13,000 x g for 5 min. A 50 μL aliquot was collected, sonicated and used for intracellular protein quantification. The remaining sample was re-suspended in culture medium containing 0.24 mmol/L fatty acid-free bovine serum albumin, 0.5 mmol/L L-carnitine, 20 mmol/L Hepes, 2 μCi [1-14C] palmitic acid (3.3 mCi/mmol, PerkinElmer) and transferred into test tubes that were tightly sealed with rubber caps. In each experimental set cells were pre-incubated for 30 min with the carnitine palmitoyltransferase inhibitor etomoxir (1 μmol/L) or with the AMP-kinase activator 5-aminoimidazole-4-carboxamide ribonucleotide AICAR (1 mmol/L), as negative and positive controls, respectively. After 2 h incubation at 37 °C, 0.3 mL of a 1:1 v/v phenylethylamine/methanol solution was added to each sample using a syringe, followed by 0.3 mL 0.8 N HClO4. Samples were incubated for a further 1 h at room temperature, then centrifuged at 13,000 x g for 10 min. Both the supernatants, containing 14CO2, and the precipitates, containing 14C-acid soluble metabolites (ASM), were collected. The radioactivity of each sample was counted by liquid scintillation. Results were expressed as pmol of [14CO2] or 14C-ASM/h/mg cell proteins.

### Immunofluorescence and immunohistochemistry

Immunofluorescence was performed by fixing cells with 4% PFA, permeabilizing them with 0.3% Triton X-100 and blocking with bovine serum albumin for 30 min. After blocking, cells were incubated with antibody at 1:100 at room temperature for 2 h, followed by incubation with 1:500 secondary antibodies Alexa fluor-488 and Alexa fluor-543 at room temperature for 1 h. Nuclei were stained with DAPI for 5 min. Immunohistochemistry experiments were performed on formalin-fixed, paraffin-embedded tissues using anti-IκBα or Ki-67 (Cell Signaling #9129) antibody according to manufacturer’s protocols.

### In vivo tumor

For in vivo tumor growth, 1 × 10^6^ IκBα shRNA cells were injected subcutaneously in 7-weeks-old female immunodeficient NSG mice (Charles River Laboratories). After 24 days mice were killed, and the tumors were removed and weighed. 1 × 10^5^ LLC cells in 100 μl of PBS were injected subcutaneously in 7 weeks old female syngeneic C57BL/6 mice. After 24 days mice were killed, and the tumors were removed and weighed.

### Statistical analysis

Two-tailed paired or unpaired Student’s t test was used to evaluate statistical significance: NSP > 0.05; *P < 0.05; **P < 0.01; ***P < 0.001. All mean values are expressed as SEM, as specified in figure legends, and derive from at least three independent experiments. Bioinformatics analyses and statistics were described in the Supplementary material methods.

More detailed descriptions of the experimental methods and analyses are available in supplementary material and Methods.

## Results

### Identification of NFKBIA amplification in cancer

**Fig. 1:**
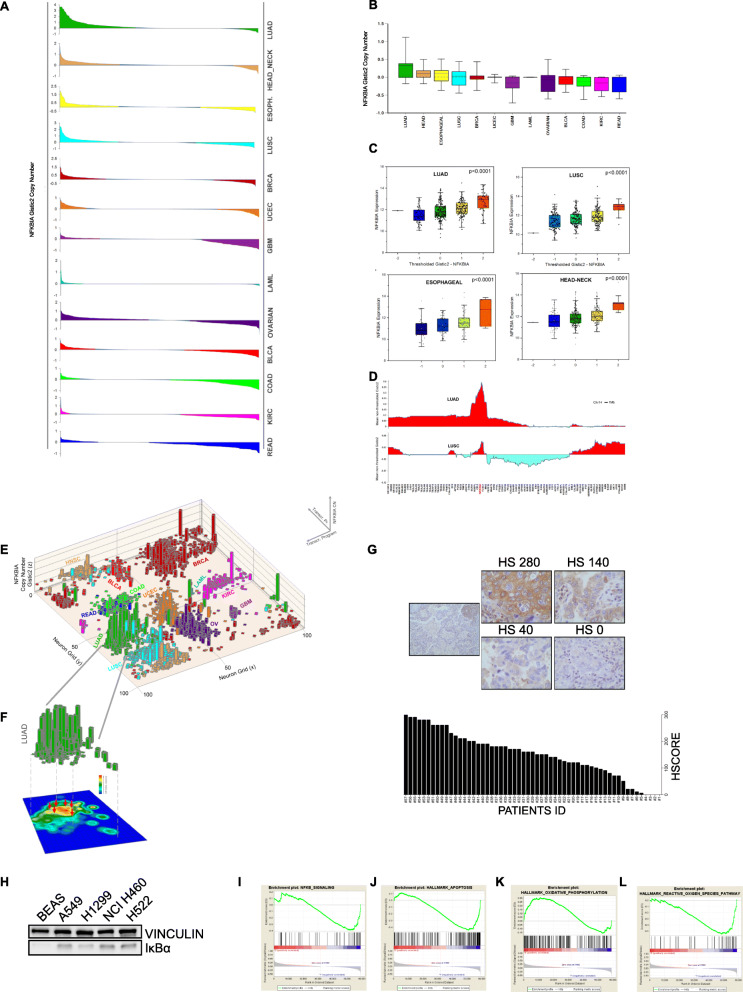
Identification of NFKBIA amplification in cancers. **a** and **b** NFΚBIA gene amplification samples analyzed by the TCGA database. Lung Adenocarcinoma (LUAD), Head and Neck Carcinoma (HEAD_NECK), Esophageal Cancer (ESOPH), Lung Squamous Carcinoma (LUSC), Breast Carcinoma (BRCA), Uterine Corpus Endometrioid Carcinoma (UCEC), Glioblastoma Multiforme (GBM), Acute Myeloid Leukemia (LAML), Ovarian Cancer (OVARIAN), Bladder Urothelial Carcinoma (BLCA), Colorectal Adenocarcinoma (COAD), Kidney Renal Clear Cell Carcinoma (KIRC), Rectal Adenocarcinoma (READ). **c** NFΚBIA mRNA expression levels analyzed by the TCGA database in Lung Adenocarcinoma, head and neck cancer, Lung Squamous Carcinoma and Esophageal Cancer. **d** Non-thresholded chromosome 14 LUAD tumors copy number profile. Positive y-axis values indicate prevalent amplification, negative values prevalent deletion. The position of a subset of chromosome 14 genes is shown in the lower part of the figure. NFΚBIA is shown in red. **e** LUAD dataset NFΚBIA copy number density heatmap. The contour of the transcriptional region characterized by the presence of high density NFΚBIA amplification is highlighted by the presence of grey arrows (**f**). **g** Representative IκBα IHC staining on lung biopsy patients. Graph showing the IHC quantification of IκBα expression in lung cancer patients. **h** Immunoblotting of IκBα and vinculin in lung cancer cells line. **i** Gene set enrichment analysis plot analyzed by the TCGA database of NF-κB signaling, apoptotic hallmarks (**j**), oxidative phosphorylation (**k**) and reactive oxygen species (**l**)

Using the Pancan12 TCGA data set [14, 15], which includes 32 studies and 10,967 cancers of different histotypes (Table S1), we measured NFKBIA (the gene coding for IκBα) copy number variations, revealing several cases of amplification in cancer. Specifically, 20.2% of Lung Adenocarcinoma (LUAD) patients displayed NFKBIA amplification, defined as a gistic2 NFKBIA copy gain ≥0.5. Similar results were obtained in Lung Squamous Cell Carcinoma (LUSC-6.5%), Esophageal Carcinoma (ESC-10.3%), Head and Neck Squamous Cell Carcinoma (HNSC-10.0%), Ovarian cancer (OV-10.0%) and Breast Invasive Carcinoma (BRCA-7.5%) (Fig. 1a and b; and Table S2). In all tested tumors, NFKBIA gene amplification is associated with increased IκBα mRNA levels (Fig. 1c). In the majority of cases the amplification of NFKBIA was a focal event (Fig. 1d and Fig. S1A), defined as an amplification smaller than a single chromosome arm [16], with the sole exception of HNSC, where the amplification often involved the entire chromosome 14. To explore the effect of NFKBIA amplification on the transcriptional program of cancer cells, an unsupervised neural network (a toroidal Kohonen Self-Organizing Map-SOMS) was initially trained using the normalized RNA-Seq data obtained from the Pancan12 dataset as input. The trained SOMS was then used to map the position of 5070 Pancan12 cancer samples on the flattened surface of the torus (x and y axes) according to their transcriptional profiles. The non-thresholded NFKBIA gistic2 copy number was plotted on the z axis. As expected, the mapping of the tumor samples was primarily influenced by the tissue of origin, as already reported in previous studies [17]. Analysis of the NFKBIA copy number density heatmap of the LUAD dataset (Fig. 1e) highlighted the presence of a well-defined transcriptional region characterized by high density of NFKBIA amplification (Fig. 1f), which suggested a potential interference of NFKBIA copy gains on the global transcriptional profile of target cells. Next, IκBα protein levels were evaluated by immunohistochemistry in a cohort of 57 lung cancers (Table S3). While in the normal lung IκBα protein expression was limited to the inflammatory tissue, where NF-κB family genes are known to be actively transcribed, the expression of IκBα protein by cancer cells was detectable in 95% of the samples with high expression in 1/3 of the samples and heterogeneous expression in the others (Fig. 1g). Expression of IκBα did not significantly change among lung cancer histotypes. To further investigate NFKBIA amplification in cancer, we profiled NFKBIA copy number in a total of 480 human cell lines. Interestingly, this analysis showed a profile very similar to the one previously identified in primary samples, with NFKBIA predominantly amplified in lung cancer, deleted in kidney and variously expressed in breast, large intestine and hematological tumors (Fig. S1B). To corroborate these observations, we also assessed IκBα protein expression by western immunoblot in various lung cancer cell lines, where IκBα appeared over-expressed when compared to normal lung cell line, as shown in Fig. 1h.

We next analyze the effect of NFKBIA amplification on the transcriptional profile of lung cancer patients. We compared the transcriptional profile of 56 NFKBIA-amplified cases (defined as samples with gistic2 score ≥ 1; 56 cases) against that of 173 NFKBIA-neutral/deleted cases (defined as gistic2 score ≤ 0). NFKBIA amplification showed an inverse correlation with p65 signature (Fig. 1i), according to the role of IκBα in preventing p65 activation. Interestingly, the transcriptional profile showed evidence of significant inverse correlation with the signature of apoptotic response associated genes (Fig. 1j), suggesting that the role of NFKBIA in cancer is far from being clarified. Moreover, the analyses showed the existence of a distinct metabolic profiles in NFKBIA amplified cancers. The most differentially expressed metabolic gene pathway in NFKBIA amplified subgroup revealed differences in mitochondrial respiration (Fig. 1k). Consistently, we observed that NFKBIA amplified cancers are also associated with a reduction in reactive oxygen species pathway (Fig. 1l). All together, these data point to unexpected metabolic role for IκBα in lung cancer.

### IκBα silencing modulates sensitivity to the DNA-damaging agent cisplatin and induces metabolic rewiring and chronic oxidative stress

**Fig. 2:**
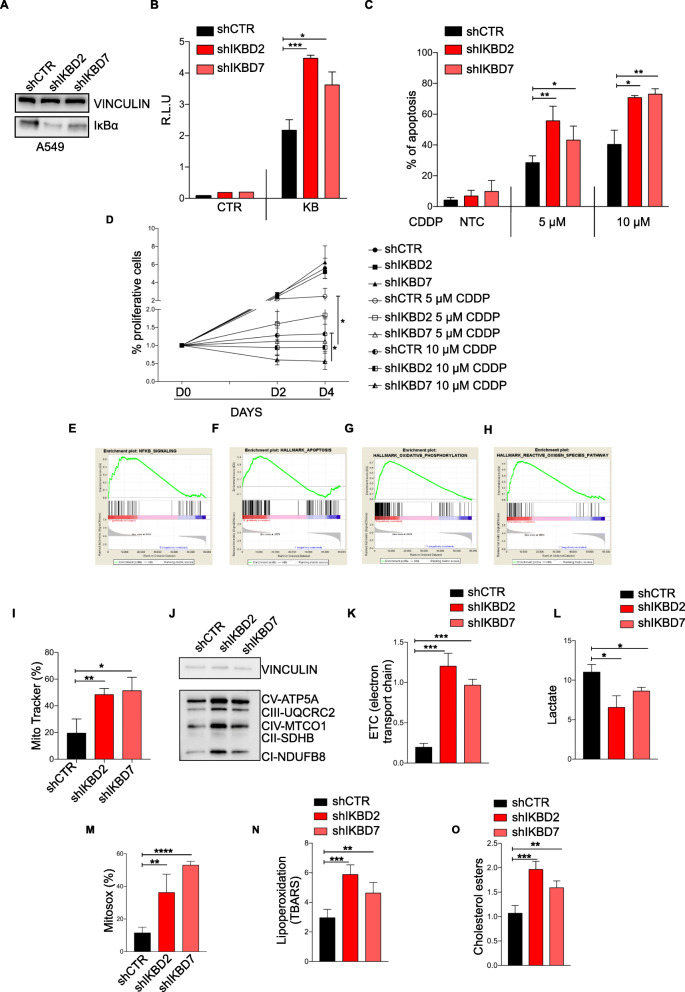
IκBα silencing modulates sensitivity to the DNA-damaging agent cisplatin and induces metabolic rewiring and chronic oxidative stress. **a** Immunoblotting of IκBα and vinculin in A549 cells infected with empty vector (shCTR) and two independent sh for IκBα (shIΚBD2 and shIΚBD7). **b** Luciferase assays of NF-κB activity in A549 cells infected with empty vector (shCTR) and two independent shIκBα (shIKBD2 and shIKBD7). Cells were transfected with NF-κB luciferase reporter or a control vector. Data are shown as mean ± sem (*n* ≥ 3 independent experiments). *P*-values are from Student’s t-test. **P < 0.01; ***P < 0.001. **c** Percentage of apoptotic A549 cells previously described in (**a**) treated with 5 μM and 10 μM cisplatin for 48 h, assessed by Annexin V. Data are shown as mean ± sem (n ≥ 3 independent experiments). *P*-values are from Student’s t-test. *P < 0.05; **P < 0.01. **d** Growth curves of A549 described in (**a**) treated with 5 μM and 10 μM cisplatin. Data are shown as mean ± sem (n ≥ 3 independent experiments). *P*-values are from Student’s t-test. *P < 0.05. **e** Gene set enrichment analysis plot of NF-κB signaling, apoptotic hallmarks (**f**) oxidative phosphorylation (**g**) and reactive-oxygen-species pathway (**h**). IκBα negatively correlated in A549 cells described in (**a**). **i** Quantification of mitochondria content by FACS analysis in A549 cells described in (**a**). Data are shown as mean ± sem (n ≥ 3 independent experiments). *P*-values are from Student’s t-test. *P < 0.05; **P < 0.01. **j** Representative Western blot showing five ETC proteins (ATP5A, ATP synthase, H+ transporting, mitochondrial F1 complex, α subunit; UQCR2, ubiquinol-cytochrome c reductase core protein II; SDHB, succinate dehydrogenase complex iron sulfur subunit B; COXII, mitochondrially encoded cytochrome c oxidase II; NDUFB8, NADH:ubiquinone oxidoreductase subunit B8) in A549 cells. I, II, III, IV, and V indicate ETC complexes. Vinculin is provided as a loading control. **k** A549 cells described in (**a**), were analyzed for ETC complexes activity, lactate production (**l**). Data are shown as mean ± sem (*n* ≥ 3 independent experiments). *P*-values are from Student’s t-test. *P < 0.05; **P < 0.01; ***P < 0.001. **m** Flow cytometry experiments measuring intracellular ROS levels in A549 cells previously described in (**a**). Data are shown as mean ± sem (n ≥ 3 independent experiments). *P*-values are from Student’s t-test. **P < 0.01; ****P < 0.0001. **n** A549 cells previously described in (**a**) were analyzed for Lipoperoxidation and cholesterol esters production (**o**). Data are shown as mean ± sem (*n* ≥ 3 independent experiments). *P*-values are from Student’s t-test. **P < 0.01; ***P < 0.001

In order to deepen the functional role of IκBα in lung cancer cell lines, we silenced this gene by two lentiviral vectors. Silenced IκBα clones (D2 and D7 in the representative A549 cell line) displayed low levels of IκBα expression (Fig. 2a) and, as expected, increased p65 transcriptional activity (Fig. 2b) compared to parental clones. Similar data were observed in the H460 cell line (Fig. S2A and B). Treatment of silenced clones with cisplatin was associated with an increased apoptosis induction (Fig. 2c and Fig. S2C) and impairment of cell proliferation (Fig. S2D) when compared to parental clones. To further investigate the role of IκBα, RNA-sequencing was performed in silenced clones and compared to the parental ones. Silenced cells showed NF-κB pathway signature (Fig. 2e) and apoptotic hallmarks (Fig. 2f) upregulation, consistent with the cisplatin-mediated increase in apoptosis. Furthermore, RNA-sequencing revealed in silenced clones an increase in transcript levels of genes involved in oxidative respiration and ROS pathway (Fig. 2g and h), suggesting a susceptibility to oxidative damage. IκBα down-modulated cells were characterized by an elevated mitochondrial content (Fig. 2i) and by the increase in Electron Transport Chain (ETC) proteins (Fig. 2j) and activity (Fig. 2k), suggesting an oxidative hypermetabolism. Interestingly, IκBα down-modulated cells showed a significant reduction in lactate production compared to controls (Fig. 2l), supporting the idea of metabolic switch from anaerobic glycolysis to oxidative metabolism. Similar data were observed in H460 cell line (Fig. S2E and F). Next, we checked reactive oxygen species (ROS) levels in IκBα down-modulated cells by mitosox and we found a strong production compared to the control (Fig. 2m). In addition, IκBα down-modulated cells exhibited more lipid oxidation products (Fig. 2n, o and Fig. S2G and H), which are further augmented upon cisplatin treatment (Fig. S3A and B) thereby confirming the role in chronic oxidative stress genesis.

Importantly, treatment with N-acetylcystein (NAC) of silenced cells is associated with a significant reduction in apoptosis induction by cisplatin, suggesting that ROS production is involved in the mediation of chemosensitivity (Fig. S3C and D).

To corroborate these observations, IκBα silenced and parental A549 cells were injected subcutaneously into NOD SCID gamma (NSG) mice (Fig. S4A). After 12 days of growth cisplatin was intraperitoneally administrated for 21 days. This approach confirmed the increased chemosensitivity of IκBα silenced cells (Fig. S4B).

Accordingly, in mice model previously described we observed that IκBα silencing is associated with changes in cellular morphology of the tumors (Fig. S4C). Consistently with these observations, silenced IκBα cells exhibit an increased in beta-oxidation as measured by functional analysis (Fig. S4D) and an up-regulation of several key lipid metabolism genes identified by RNA-sequencing analysis (Fig. S4E). Importantly, these results were validated in an independent cohort of TCGA lung cancer, observing an inverse correlation between β-oxidation signature and NFKBIA levels (Fig. S4F).

These findings reveal for the first time a strong connection between IκBα and metabolism alteration in lung cancer cells.

### IκBα-mediated metabolic rewiring and apoptotic responses through p65-NF-κB

**Fig. 3:**
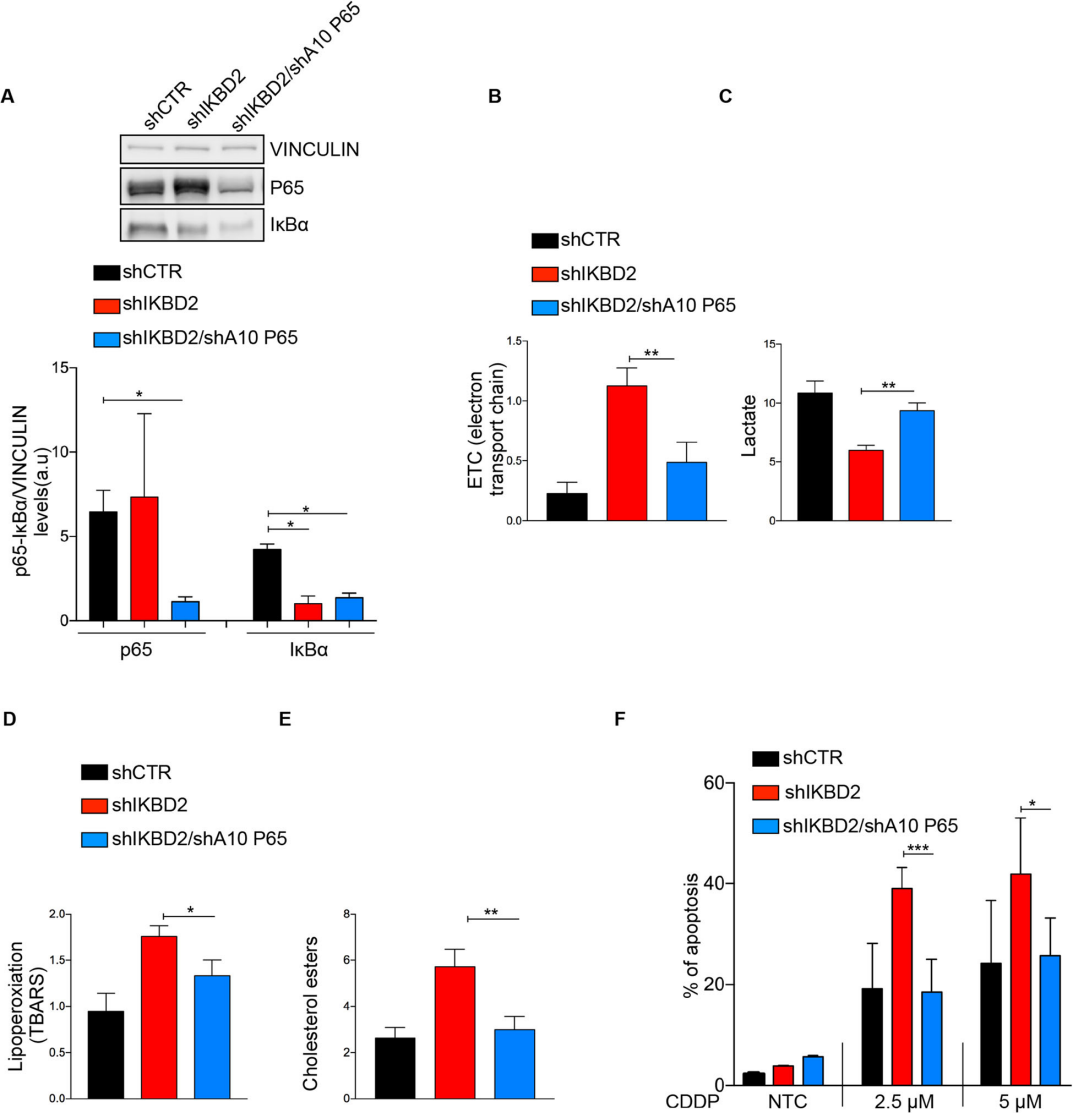
IκBα-mediated metabolic rewiring and apoptotic responses through p65-NF-κB. **a** Upper panel: Western blot analysis of extracts from A549 cells infected with shCTR, shIΚBD2 and shA10 p65 immunostained for IκBα, p65 and vinculin as a loading control; Lower pane: quantification of p65/IκBα protein levels. Data are shown as mean ± sem (*n* ≥ 3 independent experiments). *P*-values are from Student’s t-test. *P < 0.05. **b** A549 cells infected described in (**a**), were analyzed for ETC complexes activity, lactate production (**c**), lipoperoxidation (**d**) and cholesterol esters production (**e**). Data are shown as mean ± sem (n ≥ 3 independent experiments). *P*-values are from Student’s t-test. *P < 0.05; **P < 0.01. **f** Percentage of apoptosis in A549 cells previously described in (**a**) subjected for 48 h to cisplatin treatment, assessed by Annexin V. Data are shown as mean ± sem (n ≥ 3 independent experiments). *P*-values are from Student’s t-test. *P < 0.05; ***P < 0.001

Next, we aimed to define the molecular players involved in metabolic rewiring regulation and oxidative stress. To assess whether IκBα modulation acts through NF-κB, we silenced p65 expression in IκBα down-modulated cells (Fig. 3a). The metabolic changes induced by IκBα-silencing: ETC hyperactivity (Fig. 3b), drop of lactate (Fig. 3c) and lipid oxidation products accumulation (Fig. 3d and e) were at least in part rescued by p65 knock-down. All these effects participated in lowering the ROS content of cancer cells and restoring their cisplatin resistance to basal levels (Fig. 3f). Definitely, our data suggest that IκBα inhibition plays a critical role in the chemotherapy response by modulating the transcriptional activity of p65 and thereby regulating metabolism and energetic stress.

### Identification of novel small molecular compounds targeting IκBα and modulate ROS

**Fig. 4:**
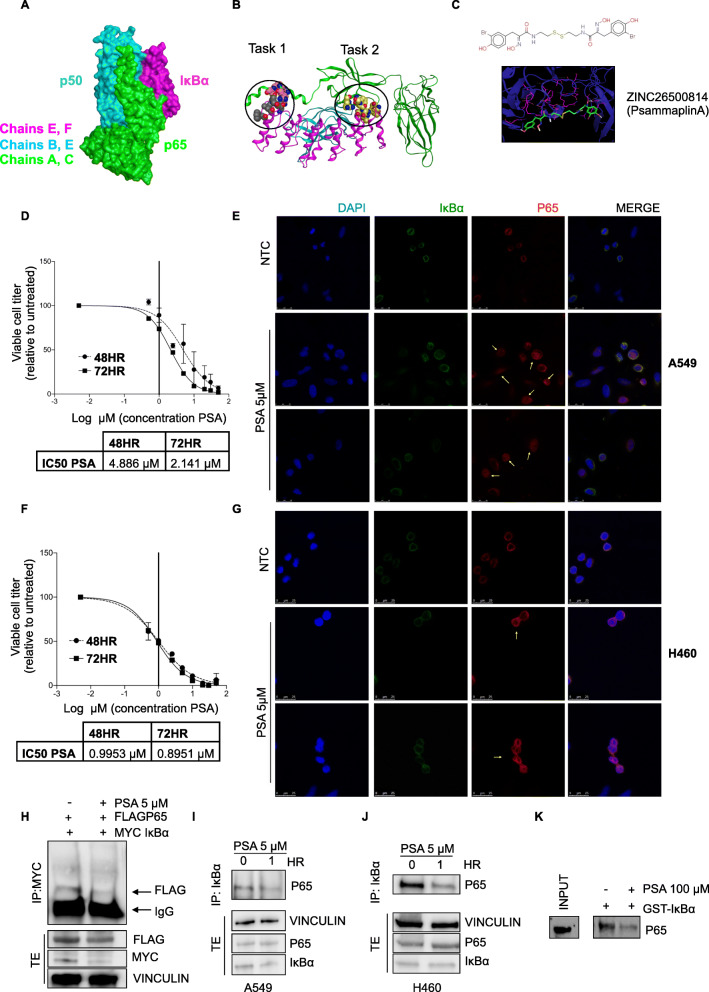
Identification of novel small molecular compounds targeting IκBα and modulate ROS. **a** Space filling representation of the ankyrin repeat domain (in magenta) bound to a partially truncated NF-κB heterodimer (p50/p65, in cyan and in green respectively), pdb code 1NFI. **b** 3D structure of the IκBα and p65 interaction with the identification of the pocket binding domain. **c** Best pose of ZINC26500814 (Psammaplin-A) in the site of binding located in the task1 of p65. **d** IC-50 values of PSA in A549 cells lines treated for 48 h and 72 h and analyzed by CTG assay. **e** Immunofluorescence on A549 cells treated with 5 μM PSA for 1 h and 2 h was performed to detect localization of endogenous p65 (red signal) and IκBα (green signal). Nuclei were stained with DAPI (blue). Yellow arrows indicate changing p65 localization. **f** IC-50 values of PSA in H460 cells lines treated for 48 h and 72 h and analyzed by CTG assay. **g** Immunofluorescence on H460 cells treated with 5 μM PSA for 1 h and 2 h was performed to detect localization of endogenous p65 (red signal) and IκBα (green signal). Nuclei were stained with DAPI (blue). Yellow arrows indicate changing p65 localization. **h** Immunoprecipitation of MYC from HEK293T transfected with a MYC-IκBα and FLAG-p65; FLAG and MYC were detected by Western blotting. The IκBα/P65 inhibitor, PSA, was added to HEK293T for 1 h. Total extract (TE) was performed as a control and was immunoblotted with, FLAG, MYC and Vinculin for loading controls. **i** and **j** Immunoprecipitation of endogenous IkBα in A549 and H460 cells, untreated or treated with 5 μM PSA for 1 h; p65 was detected by Western blotting. Total extract was used as a control and was immunoblotted with, p65, IκBα and Vinculin as loading control. **k** Western blotting of GST pulldowns showing that IκBα loses its interaction with p65. A549 cells were lysed, and GST pulldown assays were carried out by incubating lysates with immobilized purified, bacterially expressed GST- IκBα in the presence or absence of 100 μM PSA for 1 h. Pull-down fractions were subjected to Western blotting using anti-p65 antibodies

Driven by promising results obtained via genetic inhibition, we developed compounds able to disrupt the interaction between IκBα and NF-κB. Firstly, the crystallographic structure of the IκB-α/NF-κB complex (pdb code: 1NFI) was used to identify the most important residues involved in the interaction between IκBα and p65 [18–20] (Fig. 4a). The IκBα/p65 contact surface was analyzed with three tools: Maestro (https://www.schrodinger.com/) was used to visualize the residues involved in the interaction; PDBSum (http://www.ebi.ac.uk/pdbsum/) a web server that provided schematic diagrams of the interactions between proteins (Fig. S5A and B); and Pocket Query (http://pocketquery.csb.pitt.edu/) allowed exploration of hot spots and anchor residues located at the protein-protein interface. The second step of the computational procedure consisted of using the 3D structure of the IκBα/p65 complex (Fig. 4b) to virtually screen 230 million compounds from the ZINC database (https://zinc15.docking.org/) [21] (Fig. S5D). Ten putative compounds for each task were selected (Fig. S5E and F). Finally, on the basis of ADME-Tox considerations, two compounds for each task were identified and submitted to the in vitro tests: ZINC639309 and ZINC3005818 for task 1; ZINC1250968 and ZINC26500814 (also known as Psammaplin-A, PSA) (Fig. 4c) for task 2. Further validation for ZINC639309 as an IκBα/p65 dimerization inhibitor in vitro and in vivo was obtained (Fig. S6A-G). Despite the effectiveness shown by the ZINC639309 due its high IC50 we have focused our further validations on the second compound: Psammaplin-A. The IC50 of Psammaplin-A (PSA) was originally measured in A549 lung cancer cell (Fig. 4d), where it appears to act in a ∼ 5 μM range of concentrations. As a simple readout of the IκBα/p65 disruption, we observed p65 translocation into the nucleus (Fig. 4e). Similar results were obtained in H460 cells (Fig. 4f and g). Notably, PSA treatment is associated with an increased p65 transcriptional activity as detected with a luciferase assay (Fig. S7A and B). In accordance, in both A549 and H460, PSA treatment induces a significant increment in the expression of several p65-target genes (Fig. S7C and D).

To test whether PSA affects IκBα/p65 dimerization, an immunoprecipitation of IκBα in myc-tagged-IκBα transfected cells was performed and p65 was detected by western blotting (Fig. 4h). Notably, treatment with PSA appeared to disrupt IκBα/p65 binding and promote the decrease of exogenous IκBα protein (Fig. 4h). Similarly, endogenous proteins were affected as well (Fig. 4i and j). NF-κB/IκBα binding has already been shown to play a dual role: while IκBα prevents shuttling of NF-κB in to the nucleus, at the same time NF-κB prevents degradation of IκBα [22, 23]. In order to establish whether PSA prevented the formation of IκBα/p65 binding in vitro, we performed a pull-down assay with IκBα purified protein and confirmed the previous results (Fig. 4k). All these results highlight the mechanism of action and efficacy of PSA in IκBα/p65 axis disruption.

### Psammaplin-A induces apoptosis via mitochondrial bioenergetics dysfunction

**Fig. 5:**
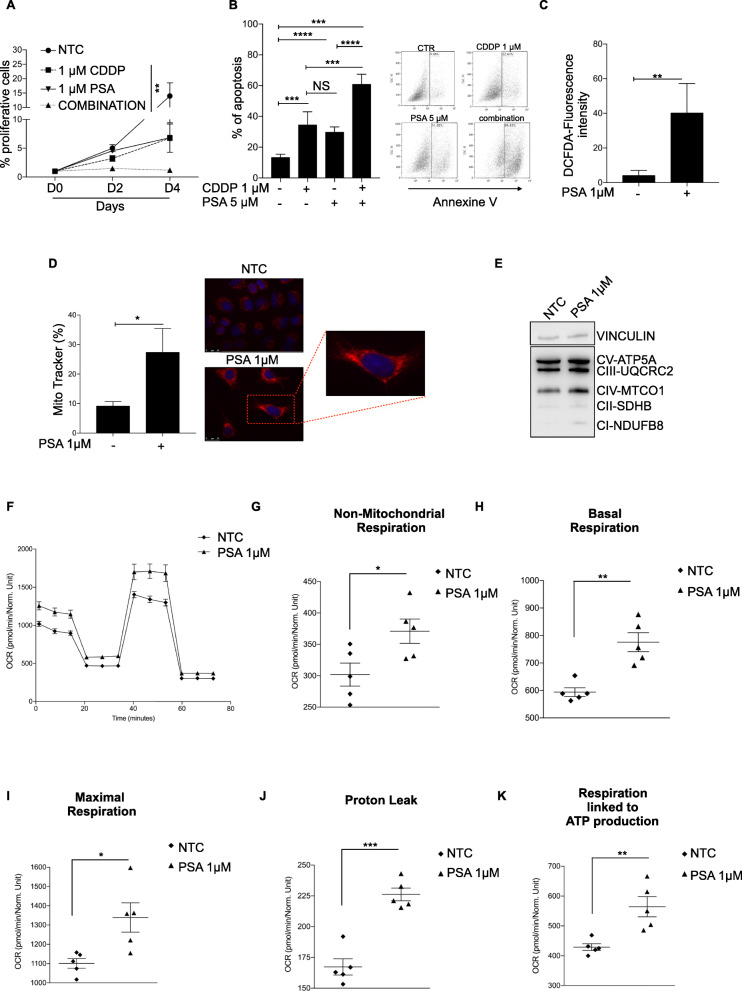
Psammaplin-A induces apoptosis via mitochondrial bioenergetics dysfunction. **a** Growth curves of A549 cells treated with 1 μM cisplatin alone or in combination with 5 μM PSA for 96 h. Data are shown as mean ± sem (*n* ≥ 3 independent experiments). *P*-values are from Student’s t-test. **P < 0.01. **b** Left panel Percentage of apoptotic A549 cells subjected to treatment with 1 μM cisplatin, 5 μM PSA or in combination with two drugs for 48 h, assessed by Annexin V; Right panel: Flow cytometry analysis of Annexine V in A549 cell line. The figure is a representative of three experiments with similar results. Data are shown as mean ± sem (n ≥ 3 independent experiments). *P*-values are from Student’s t-test. ***P < 0.001,****P < 0.0001. **c** Flow cytometry experiments measuring intracellular ROS levels in A549 cells treated with 1 μM PSA for 48 h. Data are shown as mean ± sem (n ≥ 3 independent experiments). *P*-values are from Student’s t-test. **P < 0.01. **d** Left panel: quantification of mitochondria content; Data are shown as mean ± sem (n ≥ 3 independent experiments). *P*-values are from Student’s t-test. *P < 0.05. Right panel: The representative images showed that PSA induced extensive mitochondrial fusion. **e** Representative Western blot showing five ETC proteins (ATP5A, ATP synthase, H+ transporting, mitochondrial F1 complex, α subunit; UQCR2, ubiquinol-cytochrome c reductase core protein II; SDHB, succinate dehydrogenase complex iron sulfur subunit B; COXII, mitochondrially encoded cytochrome c oxidase II; NDUFB8, NADH:ubiquinone oxidoreductase subunit B8) in A549 cells. I, II, III, IV, and V indicate ETC complexes. Vinculin is provided as a loading control. **f** Oxygen consumption rate (OCR, normalized using CyQUANT fluorescence, arbitrary units, AU). **g** Mean non-mitochondrial respiration, mean basal respiration (**h**), maximal respiration (**i**), proton leak (**j**) and respiration linked to ATP production (**k**) in cells treated with vehicle or PSA for 1 day (*n* ≥ 6). *P*-values are from Student’s t-test. ****P < 0.0001; ***P < 0.001; **P < 0.01; *P < 0.05

Next, we sought to investigate the biological effects of PSA on lung tumor cells in culture. PSA treatment of cells showing different oncogenic genotypes for 96 h was able to significantly block cancer cell proliferation alone but especially in combination with Cisplatin (Fig. 5a and Fig. S8A). In order to understand the mechanisms underlying the growth arrest, we stained the cells for Annexin V and performed a flow cytometry. We found that a 48-h treatment with PSA significantly enhanced the apoptotic effect of Cisplatin, as about 70–80% of cells displayed the apoptotic marker Annexin V (Fig. 5b and Fig. S8B). Cisplatin treatment alone induced apoptosis in 40–60% of the control cells, while cisplatin-free PSA treatment induced apoptosis in 35–60% of cells indicating the additive and combined effect of the chemotherapeutic agent and PSA on the apoptotic process. All these results indicate that PSA treatment enhances apoptotic sensitivity to cisplatin-induced cell death. Moreover, the combination of treatment with Cisplatin and PSA exerts synergistic lethal activity in A549 and H460 cells (Fig. S9A and B). To reveal the mechanisms whereby the PSA induces major sensitivity to Cisplatin treatment we evaluate total intracellular ROS production by DCFDA assay. Our results showed a significant increase of ROS levels in PSA treated cells compared to untreated (Fig. 5c and Fig. S8C). Next, we checked whether ROS production was linked to mitochondrial alterations. Surprisingly, Mitotracker staining identified elevated mitochondrial fusions (Fig. 5d and Fig. S8D, respectively) and OXPHOS complexes up-regulation following PSA treatment (Fig. 5e and Fig. S8E, respectively). In this respect, we have evaluated the expression of mitochondrial fusion markers Mitofusin 1 (MFN1), mitofusin 2 (MFN2) and optic atrophy 1 (OPA1) [24] and at various levels. In silico, using TCGA database, we observed an inverse correlation between NFKBIA levels and MFN1, OPA1 levels, while MFN2 displayed no differences (Fig. S10A, B and C). In silenced A549 and H460 clones, we observed a significant increase in MFN1 mRNA levels (Fig. S10D and E), which is in agreement with data of amplification of NFKBIA. Similarly, in both cell lines, PSA treatment favors the same increase in MFN1 mRNA levels (Fig. S10F and G) and proteins (Fig. S10H and I). All together, these data suggested an oxidative stress related to mitochondrial dysfunction, potentially linked to oxidative stress.

Finally, to confirm these findings, we assessed mitochondrial activity by measurement of the oxygen consumption rate (OCR) in A549 and H460 cells following PSA treatment for 24 h. Overall, we observed a significant increase in several parameters of mitochondrial activity including basal, maximal, and proton leak (Fig. 5f-j and Fig. S8F-J). Interestingly, PSA treatment increased the non-mitochondrial oxygen consumption (Fig. 5g), a parameter that is linked to generation of superoxide and hydrogen peroxide [25]. Although PSA induced uncoupling process in both A549 and H460 cell lines, we observed a reduced ATP-linked respiration in H460 cells (Fig. S8K) in A549 treated cells (Fig. 5k), probably due to a compensation effect linked to a different metabolic plasticity. All together our data show that PSA treatment alters mitochondrial functionality leading to a larger proton leak and ROS production. The larger proton leak is detrimental to mitochondria, since drives mitochondrial swelling and mediates cellular apoptosis, which possibly contributes to cisplatin sensitivity.

### Psammaplin-A enhances cisplatin-induced cell death in vivo

**Fig. 6:**
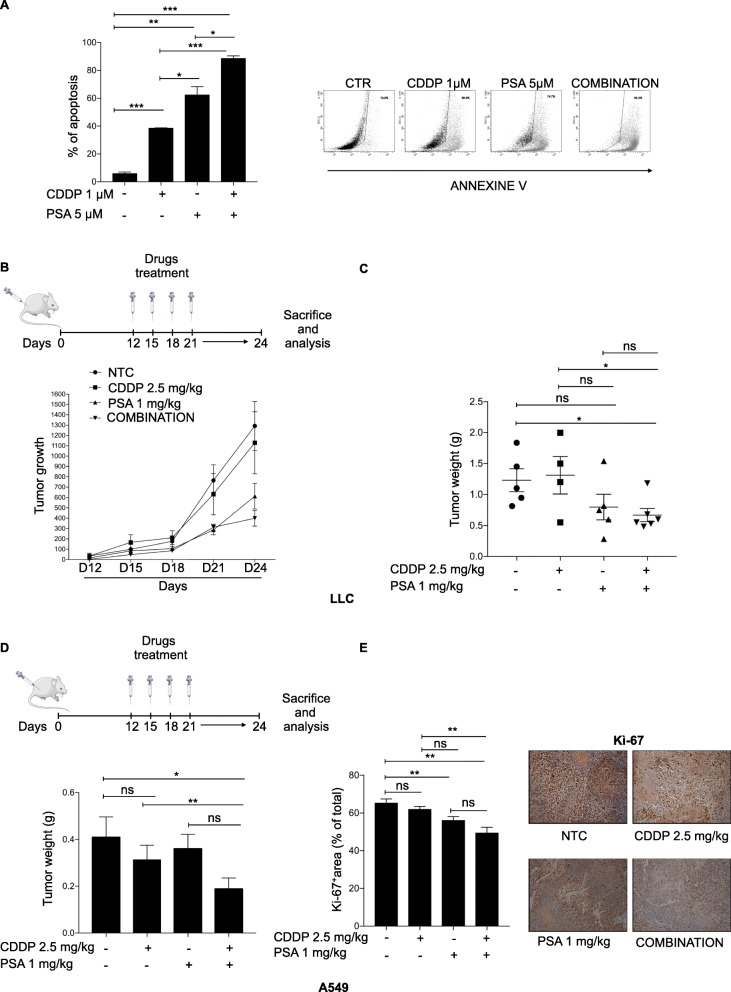
Psammaplin-A enhances cisplatin-induced cell death in vivo. **a** Left panels Percentage of apoptotic LLC cells subjected to treatment with 1 μM cisplatin, 5 μM PSA or in combination with two drugs for 48 h, assessed by Annexin V Data are shown as mean ± sem (n ≥ 3 independent experiments). *P*-values are from Student’s t-test. *P < 0.05; **P < 0.01; ***P < 0.001; Right panel: Flow cytometry analysis of Annexine V in LLC cell line. The figure is a representative of three experiments with similar results. **b** Upper panel: Schematic model of the mice treatment; Lower panel: Effects on the growth of tumors derived from subcutaneous injection of LLC cells in C57BL/6 mice treated with 2.5 mg/kg cisplatin, 1 mg/kg PSA, alone or in combination. **c** Tumor weight graph after 24 days from subcutaneous injection of LLC cells in C57BL/6 mice treated with 2.5 mg/kg cisplatin, 1 mg/kg, alone or in combination. Data are shown as mean ± sem (n ≥ 3 independent experiments). *P*-values are from Student’s t-test. *P < 0.05. **d** Upper panel: Schematic model of the mice treatment; Tumor weight graph after 24 days from subcutaneous injection of A549 cells in NSG mice treated with 2.5 mg/kg cisplatin, 1 mg/kg PSA, alone or in combination. Data are shown as mean ± sem (n ≥ 3 independent experiments). *P*-values are from Student’s t-test. **P < 0.01. **e** Quantification and representative Ki67 IHC staining. Data are shown as mean ± sem (n ≥ 3 independent experiments). *P*-values are from Student’s t-test. *P < 0.05; ***P < 0.001

In perspective of translating the intriguing in vitro results on murine models of cancer, we tested the effects of PSA alone or in combination with Cisplatin on murine Lewis lung carcinoma cell line (LLC). Also, in this model we found a strong proapoptotic effect especially with the combination (Fig. 6a) and we found that IκBα silencing affects cisplatin induced apoptosis (Fig. S11A). Next, we injected subcutaneously LLC cells into C57BL/6 J mice and measured the tumor growth after intra-peritoneal administration of the PSA and cisplatin at 1 mg/kg and 2.5 mg/kg, respectively. Coherently with in vitro data we found a significant antitumoral effect of PSA, especially in combination with cisplatin (Fig. 6b and c). PSA was already tested on mice in terms of toxicity [26] and no adverse effects were highlighted. Surprisingly, PSA in combination did not evoke toxicity in hematopoietic lineages (Fig. S11B), did not cause body weight loss ≥20% (Fig. S11C) and inhibited tumor growth even in the presence of suboptimal cisplatin concentrations. Finally, to further corroborate our findings in vivo we injected subcutaneously A549 cell line in NOD-SCID mice and we demonstrated the strong efficacy of PSA combined with low dose cisplatin on tumor growth (Fig. 6d), as also highlighted by reduced Ki67 staining (Fig. 6e). All together these results highlight the antitumoral efficacy of PSA in vivo to such an extent that we can reduce cisplatin doses.

## Discussion

Alteration of redox homeostasis are extensively studied in the field of chemotherapy since tumor cells can be effectively killed by inducing an increase in oxidative stress [27]. Above a certain threshold, acute and high concentrations of ROS alter cellular organelles and macromolecules, thus inducing cell death [28, 29]. The regulation of intracellular ROS levels is indeed of great importance in cancer treatment, because the response to commonly used chemotherapy drugs, such as cisplatin and radiotherapy agents, is directly or indirectly influenced by ROS production [30, 31].

Here, we have demonstrated that variations in IκBα levels are associated with metabolic reprogramming in lung cancer cells: high IκBα expression identifies a group of cancers with low levels of ROS and low mitochondrial oxidative metabolism, while low IκBα expression drives metabolic switch characterized by increased fatty acid oxidation, OXPHOS, mitochondrial respiration and expression of ETC components and higher levels of ROS. Our data also established a novel role of IκBα on OXPHOS patterns and eventually linked it to oxidative stress-induced chemosensitivity. Low ROS levels observed in IκBα over-expressing lung tumors correlated with a state of chemoresistance of the tumors. Conversely, the association between IκBα silencing and metabolic switching to OXPHOS could represent a therapeutic window for ROS-producing agents or oxytosis activators. Indeed, we showed that lung cancer cells silenced for IκBα, exhibited characteristics of chronic oxidative stress such as ROS content, lipid peroxides, cholesterol esters that could explain, at least in part, the enhanced chemosensitivity to Cisplatin, potentially also through oxytosis [32]. In addition, we showed that p65 is the major actor of the IκBα -mediated metabolic reprogramming of the cells, although the contribution of other IκBα partners could not be ruled out with our data. Evidence exists associating p65 with the regulation of mitochondrial function in some cancer cell types [33–35]. Therefore, it is plausible that p65 could directly regulate genes involved in oxidative phosphorylation, such as electrons transport chain or mitochondrial uncoupling (UCP) genes. In accordance, we here showed that the IκBα/p65 pair is involved in the tight regulation of two mitochondrial related genes, MFN1 and OPA1, suggesting that p65 regulates nuclear transcription of mitochondrial related genes or could have a direct role at the mitochondria [36].

Finally, following these considerations, we have proposed a novel strategy that would mimic the phenotype of IκBα silencing, through the inhibition of IκBα/p65 interaction. This approach is new to our knowledge that can push over the OXPHOS in cancer cells, resulting in increased oxidative burst and ROS production. Since cisplatin is able to shift cancer cell metabolism from glycolysis to oxidative phosphorylation [37], the perturbation of oxidative metabolism mediated by Psammaplin-A, could be the keystone for the strong efficacy in vivo and for the increased chemosensitivity. It is worth mentioning that Psammaplin-A was also shown to affect DNA replication [38], with the inhibition of histone deacetylases [38–40], histone methyltransferases [41]. However, the cytoxocicity of Psammaplin-A recapitulates the observations we obtained with IκBα silencing experiments in vitro and in vivo, therefore corroborating the utility of IκBα/NFκB targeting.

## Conclusion

The genetic or pharmacological targeting of IκBα protein in lung cancer triggers mitochondrial dysregulation and supports oxidative burst with consequent apoptosis induction. Importantly, IκBα targeting might be a novel promising therapeutical strategy to fight lung cancer.

## Supplementary Information

**Additional file 1.**

## Data Availability

Rna-seq data will be available.

## Abbreviations

NSCLC: Non-small cell lung cancer
ROS: Reactive oxygen species
OXPHOS: Oxidative phosphorylation
LUAD: Lung Adenocarcinoma
LUSC: Lung Squamous Cell Carcinoma
ESC: Esophageal Carcinoma
HNSC: Head and Neck Squamous Cell Carcinoma
OV: Ovarian cancer
BRCA: Breast Invasive Carcinoma
OCR: Oxygen consumption rate
NAC: N-acetylcystein
ETC: Electron Transport Chain

## References

[1] Herbst RS, Morgensztern D, Boshoff C (2018). The biology and management of non-small cell lung cancer. Nature.

[2] Kartalou M, Essigmann JM (2001). Mechanisms of resistance to cisplatin. Mutat Res.

[3] Kim SJ, Kim HS, Seo YR (2019). Understanding of ROS-inducing strategy in anticancer therapy. Oxidative Med Cell Longev.

[4] Kim B, Song YS (2016). Mitochondrial dynamics altered by oxidative stress in cancer. Free Radic Res.

[5] Dan Dunn J, Alvarez LA, Zhang X, Soldati T (2015). Reactive oxygen species and mitochondria: a nexus of cellular homeostasis. Redox Biol.

[6] Wang C, Youle RJ (2009). The role of mitochondria in apoptosis. Annu Rev Genet.

[7] Srinivas US, Tan BWQ, Vellayappan BA, Jeyasekharan AD (2019). ROS and the DNA damage response in cancer. Redox Biol.

[8] Saed GM, Diamond MP, Fletcher NM (2017). Updates of the role of oxidative stress in the pathogenesis of ovarian cancer. Gynecol Oncol.

[9] Raza MH, Siraj S, Arshad A, Waheed U, Aldakheel F, Alduraywish S (2017). ROS-modulated therapeutic approaches in cancer treatment. J Cancer Res Clin Oncol.

[10] Hayden MS, Ghosh S (2004). Signaling to NF-kappaB. Genes Dev.

[11] Hayden MS, Ghosh S (2012). NF-κB, the first quarter-century: remarkable progress and outstanding questions. Genes Dev.

[12] Zhang Q, Lenardo MJ, Baltimore D (2017). 30 years of NF-κB: a blossoming of relevance to human pathobiology. Cell..

[13] Mauro C, Leow SC, Anso E, Rocha S, Thotakura AK, Tornatore L (2011). NF-κB controls energy homeostasis and metabolic adaptation by upregulating mitochondrial respiration. Nat Cell Biol.

[14] Cerami E, Gao J, Dogrusoz U, Gross BE, Sumer SO, Aksoy BA (2012). The cBio cancer genomics portal: an open platform for exploring multidimensional cancer genomics data. Cancer Discov.

[15] Gao J, Aksoy BA, Dogrusoz U, Dresdner G, Gross B, Sumer SO (2013). Integrative analysis of complex cancer genomics and clinical profiles using the cBioPortal. Sci Signal.

[16] Mermel CH, Schumacher SE, Hill B, Meyerson ML, Beroukhim R, Getz G (2011). GISTIC2.0 facilitates sensitive and confident localization of the targets of focal somatic copy-number alteration in human cancers. Genome Biol.

[17] Martínez E, Yoshihara K, Kim H, Mills GM, Treviño V, Verhaak RGW (2015). Comparison of gene expression patterns across 12 tumor types identifies a cancer supercluster characterized by TP53 mutations and cell cycle defects. Oncogene.

[18] Chen FE, Huang DB, Chen YQ, Ghosh G (1998). Crystal structure of p50/p65 heterodimer of transcription factor NF-kappaB bound to DNA. Nature.

[19] Huxford T, Huang DB, Malek S, Ghosh G (1998). The crystal structure of the IkappaBalpha/NF-kappaB complex reveals mechanisms of NF-kappaB inactivation. Cell.

[20] Zheng C, Yin Q, Wu H (2011). Structural studies of NF-κB signaling. Cell Res.

[21] Irwin JJ, Shoichet BK (2005). ZINC--a free database of commercially available compounds for virtual screening. J Chem Inf Model.

[22] Mathes E, O’Dea EL, Hoffmann A, Ghosh G (2008). NF-κB dictates the degradation pathway of IκBα. EMBO J.

[23] Scott ML, Fujita T, Liou HC, Nolan GP, Baltimore D. The p65 subunit of NF-kappa B regulates I kappa B by two distinct mechanisms. Genes Dev. 1993;7(7A):1266–76. 10.1101/gad.7.7a.1266. 10.1101/gad.7.7a.1266 8319912

[24] Koshiba T, Detmer SA, Kaiser JT, Chen H, McCaffery JM, Chan DC (2004). Structural basis of mitochondrial tethering by mitofusin complexes. Science.

[25] Chacko BK, Zhi D, Darley-Usmar VM, Mitchell T (2016). The bioenergetic health index is a sensitive measure of oxidative stress in human monocytes. Redox Biol.

[26] Kim HJ, Kim TH, Seo WS, Yoo SD, Kim IH, Joo SH (2012). Pharmacokinetics and tissue distribution of psammaplin A, a novel anticancer agent, in mice. Arch Pharm Res.

[27] Ayer A, Gourlay CW, Dawes IW (2014). Cellular redox homeostasis, reactive oxygen species and replicative ageing in Saccharomyces cerevisiae. FEMS Yeast Res.

[28] Emanuele S, D’Anneo A, Calvaruso G, Cernigliaro C, Giuliano M, Lauricella M (2018). The double-edged sword profile of redox signaling: oxidative events as molecular switches in the balance between cell physiology and cancer. Chem Res Toxicol.

[29] Cui Q, Wang J-Q, Assaraf YG, Ren L, Gupta P, Wei L (2018). Modulating ROS to overcome multidrug resistance in cancer. Drug Resist Updat.

[30] de Sá Junior PL, Câmara DAD, Porcacchia AS, Fonseca PMM, Jorge SD, Araldi RP (2017). The roles of ROS in cancer heterogeneity and therapy. Oxidative Med Cell Longev.

[31] Quintiliani M (1979). Modification of radiation sensitivity: the oxygen effect. Int J Radiat Oncol Biol Phys.

[32] Tan S, Schubert D, Maher P (2001). Oxytosis: a novel form of programmed cell death. Curr Top Med Chem.

[33] Nisr RB, Shah DS, Ganley IG, Hundal HS (2019). Proinflammatory NFkB signalling promotes mitochondrial dysfunction in skeletal muscle in response to cellular fuel overloading. Cell Mol Life Sci.

[34] Guseva NV, Taghiyev AF, Sturm MT, Rokhlin OW, Cohen MB (2004). Tumor necrosis factor-related apoptosis-inducing ligand-mediated activation of mitochondria-associated nuclear factor-kappaB in prostatic carcinoma cell lines. Mol Cancer Res.

[35] Cogswell PC, Kashatus DF, Keifer JA, Guttridge DC, Reuther JY, Bristow C (2003). NF-kappa B and I kappa B alpha are found in the mitochondria. Evidence for regulation of mitochondrial gene expression by NF-kappa B. J Biol Chem.

[36] Cogswell PC, Kashatus DF, Keifer JA, Guttridge DC, Reuther JY, Bristow C (2003). NF-κB and IκBα are found in the mitochondria: evidence for regulation of mitochondrial gene expression by NF-κB. J Biol Chem.

[37] Menga A, Palmieri EM, Cianciulli A, Infantino V, Mazzone M, Scilimati A (2017). SLC25A26 overexpression impairs cell function via mtDNA hypermethylation and rewiring of methyl metabolism. FEBS J.

[38] Jiang Y, Ahn E-Y, Ryu SH, Kim D-K, Park J-S, Yoon HJ (2004). Cytotoxicity of psammaplin A from a two-sponge association may correlate with the inhibition of DNA replication. BMC Cancer.

[39] Wen J, Bao Y, Niu Q, Liu J, Yang J, Wang W (2016). Synthesis, biological evaluation and molecular modeling studies of psammaplin A and its analogs as potent histone deacetylases inhibitors and cytotoxic agents. Bioorg Med Chem Lett.

[40] Zhang B, Shan G, Zheng Y, Yu X, Ruan Z-W, Li Y (2019). Synthesis and preliminary biological evaluation of two fluoroolefin analogs of Largazole inspired by the structural similarity of the side chain unit in Psammaplin A. Mar Drugs.

[41] Byun WS, Kim WK, Han HJ, Chung H-J, Jang K, Kim HS (2019). Targeting histone methyltransferase DOT1L by a novel Psammaplin A analog inhibits growth and metastasis of triple-negative breast cancer. Mol Ther Oncolytics.

