# Supplementary material for: IκBα targeting promotes oxidative stress-dependent cell death
Source: J Exp Clin Cancer Res. 2021 Apr 16;40:136. doi: 10.1186/s13046-021-01921-x (PMC8050912; doi:10.1186/s13046-021-01921-x)
Supplement: Supplementary file 1 — Additional file 1. [file 13046_2021_1921_MOESM1_ESM.docx]

**Supplementary Materials**

**Supplementary Materials AND METHODS**

***In-silico* strategy used to identify the putative disruptors**

The *in-silico* strategy used to find the putative inhibitors is outlined in Figure S5D. The structural information about the two tasks found through Pocket Query (Figure S5) were converted in pharmacophores and submitted to ZINCPharmer (<http://zincpharmer.csb.pitt.edu/>). ZINCPharmer is a pharmacophore search web server for screening the compounds in the ZINC database (<https://zinc15.docking.org/)> against proteins, where ZINC contains over 230 million purchasable compounds in ready-to-dock 3D format. The screening of the ZINC dataset found 1336 compounds that fit Task 1 (dataset 1) and 281 that fit Task 2 (dataset 2). Dataset 1 and dataset 2 were separately imported in FLAP (v. 2.2.1, [www.moldiscovery.com](http://www.moldiscovery.com)) to refine the screening. FLAP is a software that can carry out a screening of a set of ligands against proteins using an unsupervised pharmacophoric approach based on Molecular Interaction Fields (MIFs). MIFs are calculate using the GRID force field (Goodford, 1985) that was tailored to describe non-covalent interactions between proteins and small molecules. Task 1 and Task2 were defined in FLAP (Pocket Point Radius = 2 Å) and MIFs with the default probes DRY, O, N1, and H calculated. The compounds resulting from the previous step were submitted to FLAP in their ionization state at pH = 7. FLAP generates 25 low energy conformers for each compound. For each of these conformations, the molecular interaction fields (MIFs) for H, O, N1, and DRY GRID probes were calculated at a 0.75 Å grid resolution, and FLAP fingerprints were generated. The Glob-Sum similarity score was used to rank the compounds in the dataset and the 10 compounds with the highest score were retained for each task.

**Molecular Dynamics simulations**

Task 1 was located in a very flexible region of p65, thus the two potential disruptors resulting from screening procedure were submitted to a Molecular Dynamics (MD) simulation to prove the stability of the binding for high Glob-Sum values. The small molecules identified as putative disruptors for Task 1 were firstly minimized with semi-empirical PM6 Hamiltonian using MOPAC2012 (MOPAC2012, <http://openmopac.net/> MOPAC2012.html). Then, the small molecules and the p65 protein system were prepared using BiKi Life Science tools (BiKi, ver. 1.3.5, http:// [www.bikitech.com/](http://www.bikitech.com/)) which provides an intuitive GUI interface to GROMACS and Amber tools. In particular, small molecules were parametrized with Antechamber software (ver. 14, <http://ambermd.org/> antechamber/ac.html) whereas MD simulations were performed with GROMACS (ver.4.6.1, http://www.gromacs.org/) package using the Amber ff14 (Maier et al., 2015) force field and charges computed with the AM1-BCC method (Jakalian et al., 2000). Water molecules were described using the TIP3P model (Jorgensen et al., 1983) as implemented in GROMACS. The simulated systems consisted of solvent molecules surrounding the complex between small molecules and p65 in a cubic box with periodic boundary conditions. The solvated systems were minimized using a steepest descent minimization (the maximum number of minimization cycles was set to 5000). Equilibration was carried out in four steps: firstly, three 100 ps NVT simulations were performed to gradually increase the temperature up to the final 300 K, then one 1 ns NPT step was carried out to allow the system to stably reach the pressure condition of 1 atm. Finally, one 50 ns MD production run was performed with a time step for integration equal to 0.002 ps and a number of steps equal to 25,000,000. Coordinates were saved every 10 ps; in total 5000 snapshots were obtained. The temperature coupling was done using a velocity rescaling with a stochastic term that ensures that a proper canonical ensemble is generated. (Bussi et al., 2007) The putative inhibitors were located in the Task 1 according to the poses found in FLAP, then a MD run was performed to verify that the binding is stable during the simulation. Figure S5 showed the results for the compounds ZINC40188517 and ZINC93795489, high and low Glob-Sum scores respectively. Potential disruptors with high Glob-Sum values is expected to form stable binding despite the flexibility of the protein, thus Glob-Sum was used as the score function in FLAP.

**ADME-Tox prioritization procedure**

An ADME-Tox filter was applied to the results of the screening procedure to prioritize the compounds before submitting to in-vitro tests. The following ADME-Tox parameters were calculated:

- Ionization: the ionization state (ne=neutral; ca=cation; an= anion; zw= zwitterionic) was calculated with MoKa (v. 3.2.1, <http://www.moldiscovery.com>) at pH=7.0. Neutral compounds were prioritized.
- Lipophility: the log P was considered as a lipophilicity index and was calculated with MoKa (v. 3.2.1, <http://www.moldiscovery.com>). According Rule of 5 (Ro5) and other drug discovery metrics, a log P greater than 5 is considered detrimental for the absorption of the candidate. Because of the uncertainty associated to log P calculation, we adopted a less stringent criteria: a log P values less than 5 were considered acceptable (green color), log P between 5 and 6 were considered not optimal (orange color), compounds with log P greater than 6 were discarded;
- HBD: the number of hydrogen-bond donor atoms was calculated with DRAGON 7 (the software was kindly provide by Prof. Todeschini). According to the Ro5 rule and other metrics, a number of HBD greater than 5 is detrimental for the absorption of the candidate. We adopted a less stringent criteria: compounds with a number of HBD less than 6 are considered acceptable (green color); compounds with HBD equal to 6 were considered not optimal and, finally, HBD numbers greater than 6 are considered detrimental (red color);
- P-glycoprotein (Pgp): the capability of the compounds to inhibit Pgp and/or to be a substrate of Pgp was predicted using the ADMETlab platform (http://admet.scbdd.com/calcpre/index/). In principle it should be better that the compounds do not interact with Pgp (green color), however in very early drug discovery Pgp interaction could be considered a moderate drawback (orange color).

**Final selection of the compounds**

Finally, the Glob-Sum score calculated by FLAP was used to rank the efficacy of the binding of the inhibitor to p65. A Glob-Sum threshold of 2 was used to identify the most promising compounds. As showed in Figure S1E and S1F, the combination of Glob-Sum and ADME-Tox criteria allowed us to directly exclude 7 compound for both task 1 and 2. For the Task 2, compounds ZINC000000639309 and ZINC03005818 showed a satisfactory GLO-Sum and the lower numbers of warning (orange color) in ADME-Tox criteria. For the Task1, ZINC01250968 was clearly identified as the most promising compounds. The choice between ZINC26500814 and ZINC08425287 was more complex because the first showed 5 warnings but a more favorable Glob-Sum values whereas the opposite is true for the second. We preferred to prioritize the candidate with the best Glob-Sum which was ZINC26500814.

**Unthresholded Gistic2 data acquisition**

Primary tumor Log2 NFKBIA Gistic2 data, generated using whole genome microarray assays at the TCGA genome characterization center, were downloaded from the https://tcga.xenahubs.net portal. The following tumors were selected: LUAD, Head and Neck, LUSC, BRCA, UCEC, GB, LAML, Ovarian, BLCA, COAD, KIRC and READ. Data were downloaded from the following links: https://tcga.xenahubs.net/download/TCGA.LUAD.sampleMap/Gistic2_CopyNumber_Gistic2_all_data_by_genes.gz (LUAD); https://tcga.xenahubs.net/download/TCGA.LUSC.sampleMap/Gistic2_CopyNumber_Gistic2_all_data_by_genes.gz (LUSC); https://tcga.xenahubs.net/download/TCGA.ESCA.sampleMap/Gistic2_CopyNumber_Gistic2_all_data_by_genes.gz (Esophageal); https://tcga.xenahubs.net/download/TCGA.HNSC.sampleMap/Gistic2_CopyNumber_Gistic2_all_data_by_genes.gz (Head and Neck); https://tcga.xenahubs.net/download/TCGA.BRCA.sampleMap/Gistic2_CopyNumber_Gistic2_all_data_by_genes.gz (BRCA); https://tcga.xenahubs.net/download/TCGA.UCEC.sampleMap/Gistic2_CopyNumber_Gistic2_all_data_by_genes.gz (UCEC); https://tcga.xenahubs.net/download/TCGA.GBM.sampleMap/Gistic2_CopyNumber_Gistic2_all_data_by_genes.gz (GBM); https://tcga.xenahubs.net/download/TCGA.LAML.sampleMap/Gistic2_CopyNumber_Gistic2_all_data_by_genes.gz (LAML); https://tcga.xenahubs.net/download/TCGA.OV.sampleMap/Gistic2_CopyNumber_Gistic2_all_data_by_genes.gz (Ovarian); https://tcga.xenahubs.net/download/TCGA.BLCA.sampleMap/Gistic2_CopyNumber_Gistic2_all_data_by_genes.gz (BLCA);https://tcga.xenahubs.net/download/TCGA.COAD.sampleMap/Gistic2_CopyNumber_Gistic2_all_data_by_genes.gz (COAD); https://tcga.xenahubs.net/download/TCGA.KIRC.sampleMap/Gistic2_CopyNumber_Gistic2_all_data_by_genes.gz (KIRC); https://tcga.xenahubs.net/download/TCGA.READ.sampleMap/Gistic2_CopyNumber_Gistic2_all_data_by_genes.gz (READ)

**Whole-transcriptome TCGA data acquisition**

Primary sample polyA+ RNA-Seq data for the following tumors: LUAD, Head and Neck, LUSC, BRCA, UCEC, GB, LAML, Ovarian, BLCA, COAD, KIRC and READ were downloaded from the TCGA repository at: https://tcga.xenahubs.net/download/TCGA.LUAD.sampleMap/HiSeqV2.gz (LUAD), https://tcga.xenahubs.net/download/TCGA.LUSC.sampleMap/HiSeqV2_exon.gz (LUSC), https://tcga.xenahubs.net/download/TCGA.ESCA.sampleMap/HiSeq_exon.gz (Esophageal), https://tcga.xenahubs.net/download/TCGA.HNSC.sampleMap/HiSeqV2.gz (Head and Neck), https://tcga.xenahubs.net/download/TCGA.BRCA.sampleMap/HiSeqV2_exon.gz (BRCA), https://tcga.xenahubs.net/download/TCGA.UCEC.sampleMap/HiSeqV2_exon.gz (UCEC), https://tcga.xenahubs.net/download/TCGA.GBM.sampleMap/HiSeqV2_exon.gz (GBM), https://tcga.xenahubs.net/download/TCGA.LAML.sampleMap/HiSeqV2_exon.gz (LAML), https://tcga.xenahubs.net/download/TCGA.OV.sampleMap/HiSeqV2_exon.gz (Ovarian), https://tcga.xenahubs.net/download/TCGA.BLCA.sampleMap/HiSeqV2_exon.gz (BLCA), https://tcga.xenahubs.net/download/TCGA.COAD.sampleMap/HiSeqV2_exon.gz (COAD), https://tcga.xenahubs.net/download/TCGA.KIRC.sampleMap/HiSeqV2_exon.gz (KIRC), https://tcga.xenahubs.net/download/TCGA.READ.sampleMap/HiSeqV2_exon.gz (READ)

**NFKBIA copy number in primary tumors**

Unthresholded NFKBIA Gistic2 data for the following tumor types: LUAD, Head and Neck, LUSC, BRCA, UCEC, GB, LAML, Ovarian, BLCA, COAD, KIRC and READ tumors were downloaded from the TCGA hub as already described. Mean NFKBIA Gistic2 data for individual tumor types were then sorted in descending order and plotted as area graph.

**NFKBIA copy number in cancer cell lines**

Mean unthresholded Gistic2 NFKBIA copy number data for lung cancer, large intestine, breast, hematopoietic neoplasms and kidney cancer cell lines were downloaded from the Xena UCSC Cancer Cell Line Encyclopedia DB by setting "!=null AND target-tissue" as filter. Gistic2 data for individual tumor cell lines were then sorted in descending order according to the mean NFKBIA copy number score and plotted as area graph.

**Chromosome 14 Gistic2 map**

Unthresholded Gistic2 data from LUAD, Head and Neck, LUSC, BRCA, UCEC, GB, LAML, Ovarian, BLCA, COAD, KIRC and READ tumors were used to extract the mean copy number level of all the genes present in chromosome 14. Gene coordinates were defined using the comprehensive gene annotation dataset of the Gencode database v.24 (https://www.gencodegenes.org/human/). Mean, per-gene log2 copy number data were plotted as area graph.

**RNA-Seq differential expression analysis of primary tumors**

LUAD differential gene expression analysis was performed on raw counts with DESeq2 (Genome Biol. 2014;15(12):550) using a ‘design = ~ condition’ linear model. The condition factor for each LUAD sample was defined as ‘0’ or ‘1’ according to the associated NFKBIA Gistic2 level. Specifically, samples with unthresholded NFKBIA Gistic2 ≥ +1 were considered as cases while samples with NFKBIA Gistic2 ≤ 0 were considered as controls. A total of 229 primary LUAD samples were analyzed. Of them 56 showed NFKBIA amplification and 173 were NFKBIA copy number neutral. The differentially expressed genes were filtered by setting the adjusted p-value (Benjamini-Hochberg False Discovery Rate - FDR) at 0.1.

Subsequent GSEA analyses were performed using the Broad Institute GSEA tool http://software.broadinstitute.org/gsea/index.jsp by selecting a phenotype permutation model with a total of 2000 permutations and the Human v6.2 hallmark gene set (gseaftp.broadinstitute.org://pub/gsea/gene_sets_final/h.all.v6.2.symbols.gmt) as reference. Significantly enriched gene sets were filtered by selecting the adjusted p-value (FDR) at 0.25, as suggested by the GSEA authors.

**Combined Copy number and whole-transcriptome analyses**

To assess if the NFKBIA expression level was different in samples with different NFKBIA mean Gistic2 scores, thresholded Gistic2 data for LUAD, Head and Neck, LUSC, BRCA, UCEC, GB, LAML, Ovarian, BLCA, COAD, KIRC and READ tumors were downloaded from the following links: https://tcga.xenahubs.net/download/TCGA.LUAD.sampleMap/Gistic2_CopyNumber_Gistic2_all_thresholded.by_genes.gz (LUAD); https://tcga.xenahubs.net/download/TCGA.LUSC.sampleMap/Gistic2_CopyNumber_Gistic2_all_thresholded.by_genes.gz (LUSC); https://tcga.xenahubs.net/download/TCGA.ESCA.sampleMap/Gistic2_CopyNumber_Gistic2_all_thresholded.by_genes.gz (Esophageal); https://tcga.xenahubs.net/download/TCGA.HNSC.sampleMap/Gistic2_CopyNumber_Gistic2_all_thresholded.by_genes.gz (Head and Neck); A One-Way ANOVA test was performed (Origin Pro 2019b) to test for differences in NFKBIA expression across groups. The statistic test was considered to be significant in presence of a p-value < 0.05.

**Whole-transcriptome dimensionality reduction using Kohonen Self-Organizing Maps**

A Kohonen Self-Organizing Map (SOM) was generated using the Encog library v. 3.4.0 (https://www.heatonresearch.com/encog/), custom-modified in order to generate toroidal maps. A total of 10000 neurons, organized in a 100x100 toroidal grid, were used as output neurons. A Gaussian radial basis function was used to calculate the training falloff, controlled by a start learning rate of 0.9, an end learning rate of 0.1, a start radius of 80 and an final radius of 5. A total of 600 iterations were run to generate the final map. After the completion of the training step, a total of 4668 tumor samples were subsequently mapped to the SOM. The position of each tumor sample, defined by its transcriptome profile, was mapped on the x,y grid using a Best Matching Unit approach. Samples mapped on the x,y grid were further annotated with unthresholded NFKBIA Gistic2 data on the z axis. Positional information specific for the LUAD samples, together with the associated NFKBIA Gistic2 data were then used to generate the 2d density map using custom software. Briefly, intensity of the Gistic2 data was initially used to create a greyscale intensity mask, with signal intensity directly proportional to the Gistic2 score after log2-to-linear conversion and modeled as a linear density gradient with a 14-pixels radius. Different signals were linearly combined and capped at 255. The greyscale intensity mask was then converted into a color heatmap using a linear blue-cyan-green-yellow-red palette with no color breaks.

**RNA-Seq lines**

Four lung A549 siNFKBIA cell lines and two scrambled A549 control lines were analyzed by RNA-Seq, with 25 Million of reads each and a paired-end strategy. Gzipped fastq sequences were aligned to the reference GRCh38.p5 Gencode human genome using STAR v. 2.5.0c and the Gencode v24 GTF annotation file with the following main parameters: outFilterMultimapNmax 20; alignSJoverhangMin 8; alignSJDBoverhangMin 1; outFilterMismatchNmax 999; outFilterMismatchNoverLmax 0.04; alignIntronMin 20; alignIntronMax 1000000; alignMatesGapMax 1000000; alignTranscriptsPerReadNmax 100000; quantMode TranscriptomeSAM. Raw counts were then analyzed in order to identify the differentially expressed genes by using DESeq2 (Genome Biol. 2014;15(12):550) with a ‘design = ~ condition’ linear model. The condition factor for each sample was defined as ‘0’ for control and as ‘1’ for siNFKBIA lines. The differentially expressed genes were filtered by setting the adjusted p-value (Benjamini-Hochberg False Discovery Rate - FDR) at 0.1. Subsequent GSEA analyses were performed using the Broad Institute GSEA tool http://software.broadinstitute.org/gsea/index.jsp by selecting a gene_set permutation model with a total of 1000 permutations and the Human v6.2 hallmark gene set (gseaftp.broadinstitute.org://pub/gsea/gene_sets_final/h.all.v6.2.symbols.gmt) as reference. Significantly enriched gene sets were filtered by selecting the adjusted p-value (FDR) at 0.25, as suggested by the GSEA authors.

**Heatmap**

The leading edge gene list for the Reactive Oxygen Species GSEA gene set of the A549 siNFKBIA RNA-Seq experiment was calculated using the GSEA tool (http://software.broadinstitute.org/gsea/index.jsp). RNA-Seq raw counts corresponding to the leading edge genes were row-scaled and plotted using the R package heatmap.2 with no color breaks.

**Statistical analysis**

Two-tailed paired or unpaired Student‘s t test was used to evaluate statistical significance: NSP>0.05; *P<0.05; **P<0.01; ***P<0.001; ****P<0.0001. All mean values are expressed as sem, as specified in figure legends, and derive from at least three independent experiment.


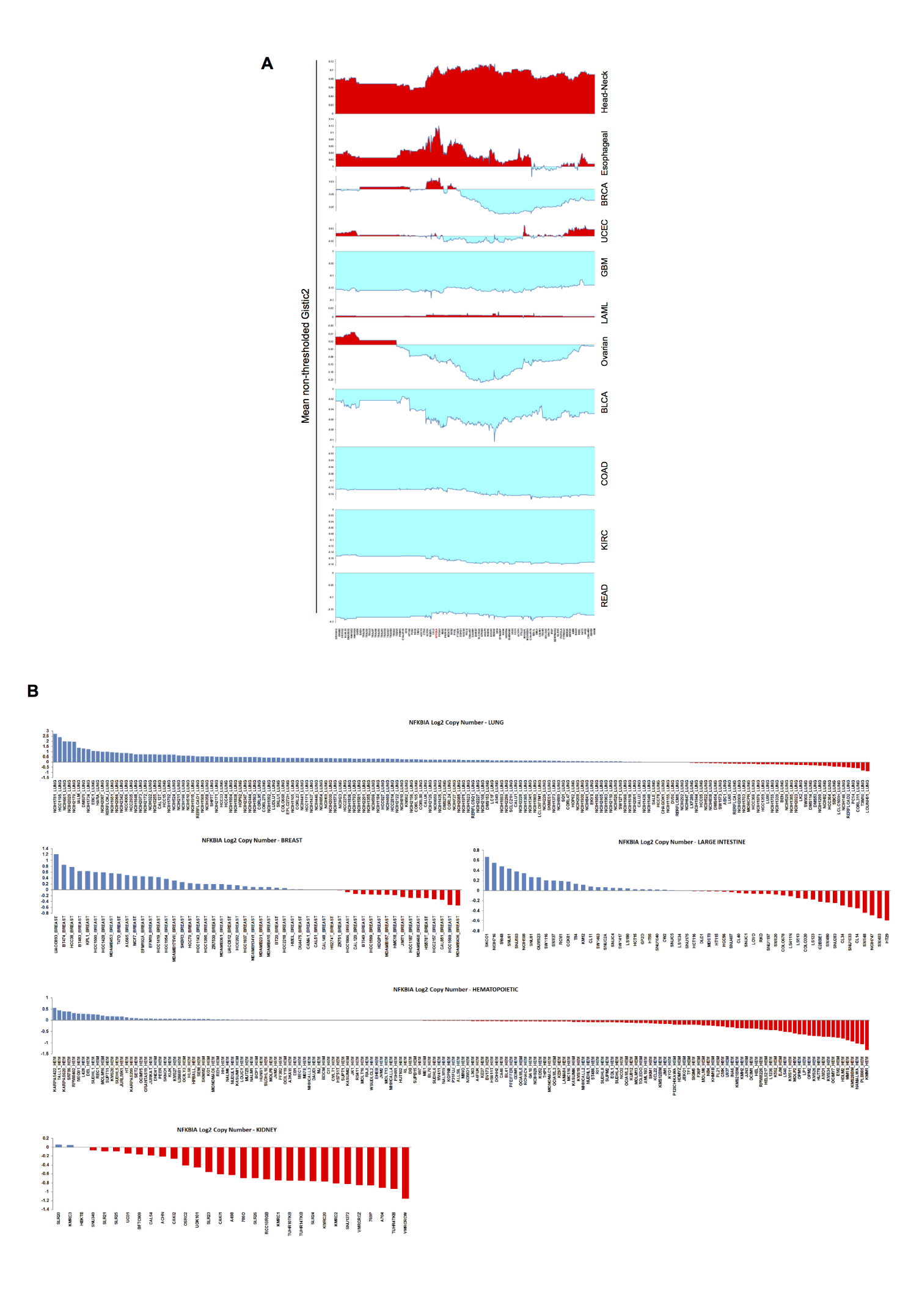
**Supplementary figures**

**Fig. S1. NFKBIA amplification profile plot in TCGA data set and copy number in cancer cell lines**

**(A)** Mean non-thresholded Gistic2 score profile plot showing NFKBIA amplification (red) or deletion (light blue). Head and Neck Carcinoma (HEAD_NECK), Esophageal Cancer (ESOPH), Breast Carcinoma (BRCA), Uterine Corpus Endometrioid Carcinoma (UCEC), Glioblastoma Multiforme (GBM), Acute Myeloid Leukemia (LAML), Ovarian Cancer (OVARIAN), Bladder Urothelial Carcinoma (BLCA), Colorectal Adenocarcinoma (COAD), Kidney Renal Clear Cell Carcinoma (KIRC), Rectal Adenocarcinoma (READ). **(B)** Histograms of NFKBIA copy number evaluation in lung, breast, large intestine, hematopoietic and kidney cancer cells lines.

**
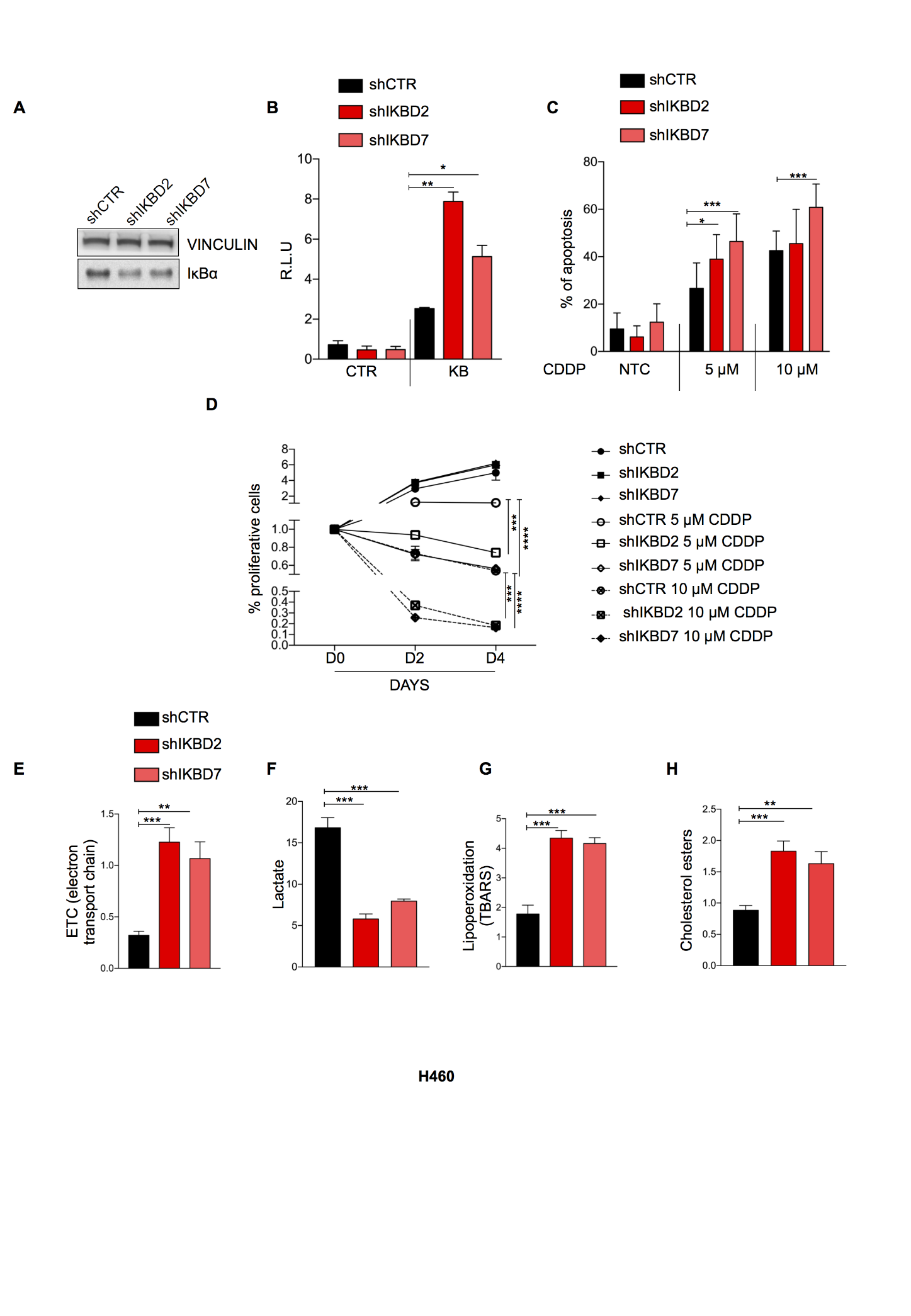
**

**Fig. S2. IκBα-silencing promotes apoptosis induction and metabolic rewiring of cancer cells**

**(A)** Western blot analysis of H460 cells infected with empty vector (shCTR) and two independent sh for IκBα (shIKBD2 and shIKBD7) immunostained IκBα and VINCULIN as loading control. **(B)** Luciferase assays of NF-κB activity in H460 cells infected with empty vector (shCTR) and two independent sh for IκBα (shIKBD2 and shIKBD7). Cells were transfected with NF-κB luciferase reporter or a control vector. Data are shown as mean ± sem (n ≥ 3 independent experiments). To evaluate if IκBα can influence NF-κB activity we used a luciferase reporter gene with specific NF-κB binding sites in the promoter region. IκBα downregulation significantly increased NF-κB activity. **(C)** Percentage of apoptotic H460 cells previously described in (A) treated with 5µM and 10µM Cisplatin for 48 h, assessed by Annexin V. Data are shown as mean ± sem (n ≥ 3 independent experiments). *P*-values are from Student’s t-test. *P<0.05; ***P<0.001 **(D)** Growth curves of H460 cells previously described in (A) and treated with 5µM and 10µM cisplatin. Data are shown as mean ± sem (n ≥ 3 independent experiments). P-values are from Student’s t-test. ***P<0.001; ****P<0.0001. **(E)** H460 cells previously described in A were analyzed for ETC complexes, lactate production **(F)**, Lipoperoxidation **(G)**, cholesterol esters production **(H).** Data are shown as mean ± sem (n ≥ 3 independent experiments). *P*-values are from Student’s t-test.**P<0.01; ***P<0.001.


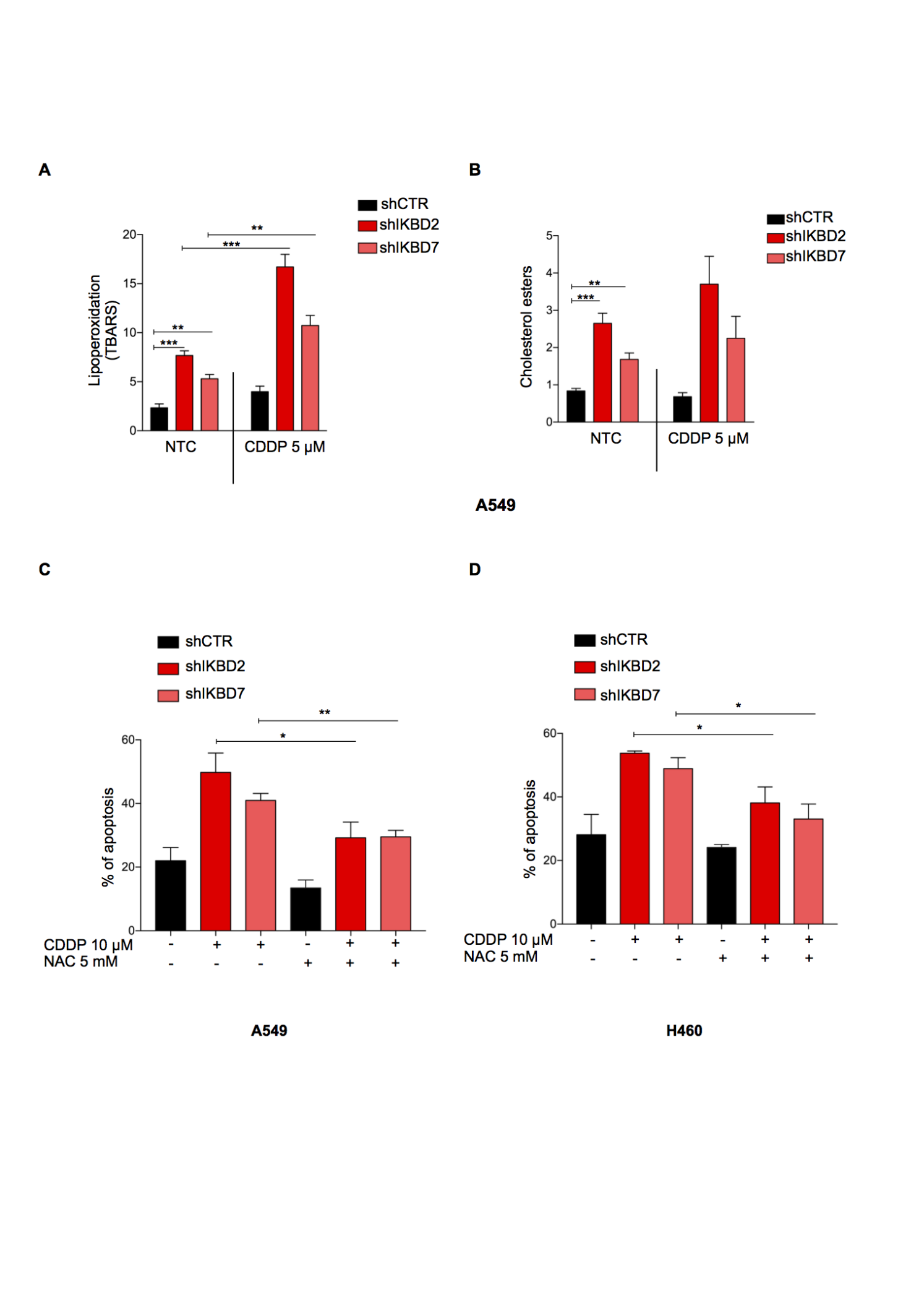


**Fig. S3. IκBα silencing modulates ROS production**

**(A)** A549 cells infected with empty vector (shCTR) and two independent sh for IκBα (shIKBD2 and shIKBD7) were analyzed for lipoperoxidation and cholesterol esters production **(B),** after 48h of 5 μM cisplatin treatment. Data are shown as mean ± sem (n ≥ 3 independent experiments). P-values are from Student’s t-test. **P<0.01; ***P<0.001. **(C)** Percentage of apoptosis in A549 cells previously described in (A) subjected for 24 h to cisplatin and NAC treatment, assessed by Annexin V. Data are shown as mean ± sem (n ≥ 3 independent experiments). P-values are from Student’s t-test. *P<0.05; **P<0.01 **(D)** Percentage of apoptosis in H460 cells infected with empty vector (shCTR) and two independent sh for IκBα (shIKBD2 and shIKBD7) subjected for 24 h to cisplatin and NAC treatment, assessed by Annexin V. Data are shown as mean ± sem (n ≥ 3 independent experiments). P-values are from Student’s t-test. *P<0.05.


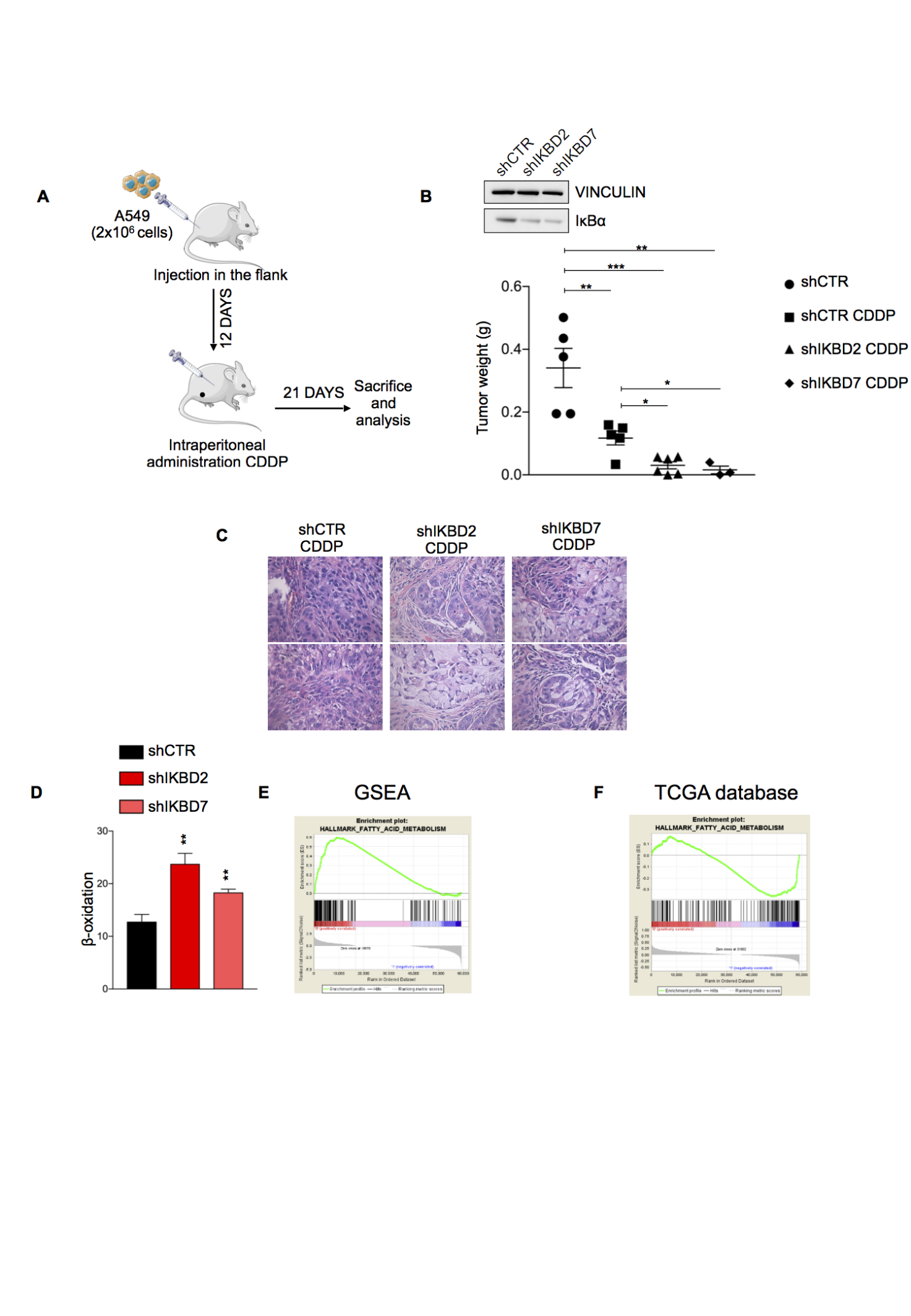


**Fig. S4. IκBα silencing promotes apoptosis  and  metabolic rewiring of cancer cells.**

**(A)** Schematic model of  the mice treatment. **(B)** Tumor weight graph after 21 day from subcutaneous injection of A549 previously described in (A) in NSG mice, treated with 5mg/kg Cisplatin. **(C)** Histologic examination of tumoral tissue samples after 21 days from subcutaneous injection of A549 previously described in (A). Hematoxylin and eosin (H&E) staining. **(D)** A549 described in A were analyzed for  β-oxidation. **(E)** Gene set enrichment analysis plot of fatty acid metabolism  IκBα-negatively correlated. **(F)** Gene set enrichment analysis plot analyzed by the TCGA database of fatty acid metabolism.


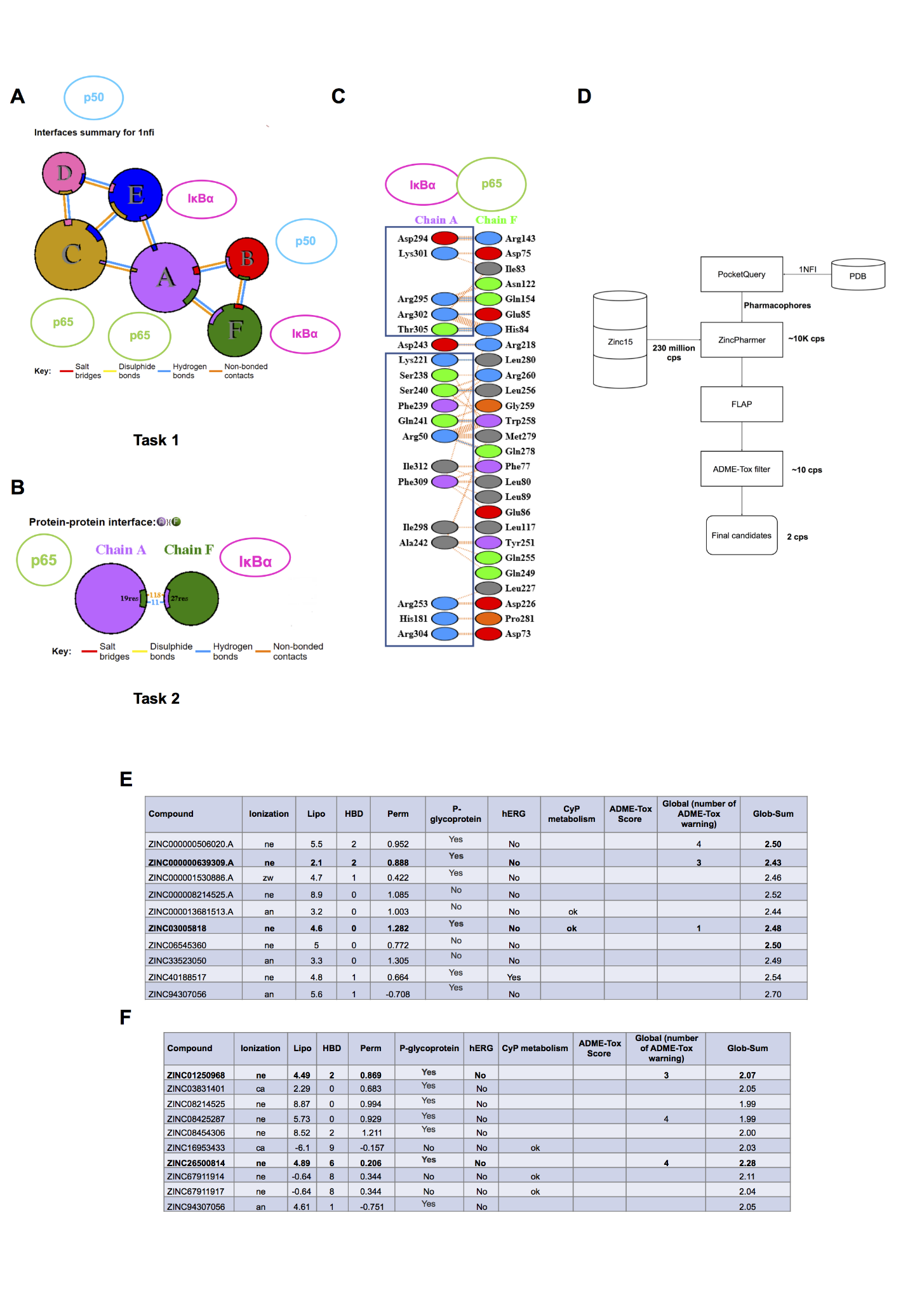


**Fig. S5. PDBSum analysis of pdb structure 1NFI and ADME_Tox predicted data and Glob-Sum**

**(A)** Diagram of the protein-protein interactions between the three proteins involved in the IκBα/NF-κB complex. **(B)** Focus on the interaction between the ankyrin repeat domain of IκBα and the p65 portion of NF-κB. **(C)** List of the main residues involved in the interaction between p65 and IκBα. Some labels were added to clarify the diagrams and to define the two pockets. **(D)** Schematic representation of strategy to find task 1 and 2. **(E)-(F)** Table with ADME_Tox predicted data and Glob-Sum.


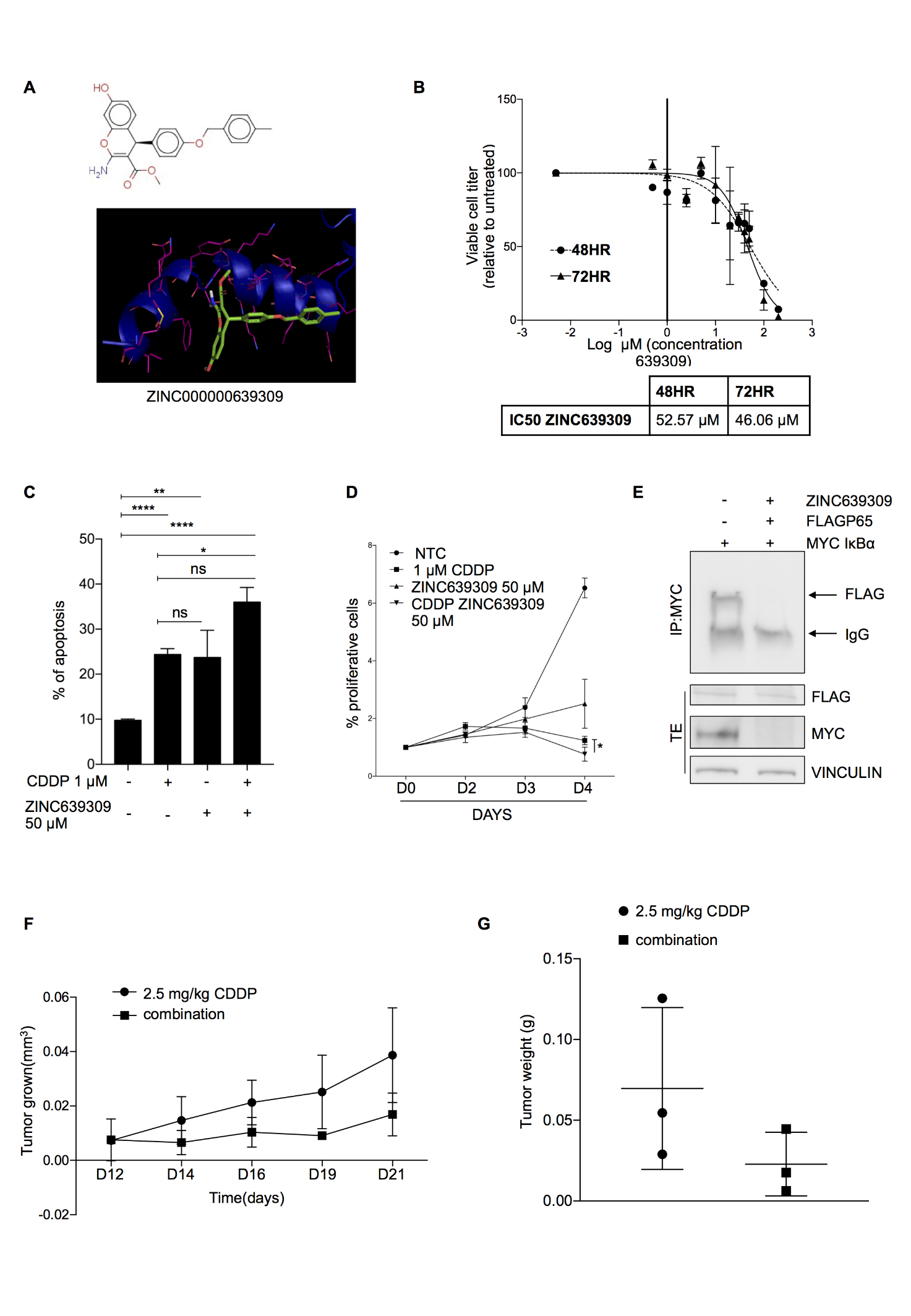


**Fig. S6. Evaluation task 1 compound**

**(A)** Best pose of ZINC000000639309 in the site of binding located in the task2 of p65. (**B)** IC-50 values of ZINC639309 in A549 cells lines treated for 48 h and 72 h and analyzed by CTG assay. **(C)** Percentage of apoptotic A549 cells subjected to treatment with 1µM Cisplatin, 50µM ZINC639309 alone or in combination for 48 h, assessed by Annexin V. Data are shown as mean ± sem (n ≥ 3 independent experiments). *P*-values are from Student’s t-test. *P<0.05; **P<0.01; ***P<0.001. **(D)** Growth curves of A549 cells treated with 1µM Cisplatin, 50µM ZINC639309 alone or in combination with for 96 h. Data are shown as mean ± sem (n ≥ 3 independent experiments). *P*-values are from Student’s t-test. *P<0.05 **(E)** Immunoprecipitation of MYC from HEK293T transfected with a MYC-IκBα and FLAG-p65; FLAG and MYC were detected by Western blot. The IκBα/p65 inhibitor, ZINC639309, was added to HEK293T for 1h. TE, total extract was performed as a control and was immunoblotted with, FLAG, MYC and VINCULIN as loading control. **(F)** Upper panel: representative images of NSG mice injected subcutaneously with 2×10^6^ A549 cells at day 12 and 24 post‐injection; lower panel: tumor growth curves starting after 12 days from subcutaneous injection of A549 cells treated with Cisplatin 2.5mg/kg or with a combination of Cisplatin and ZINC639309 10mg/kg. **(G)** Tumor weight graph after 24 days from subcutaneous injection of A549 in NGS mice (*n* = 3 mice per group).


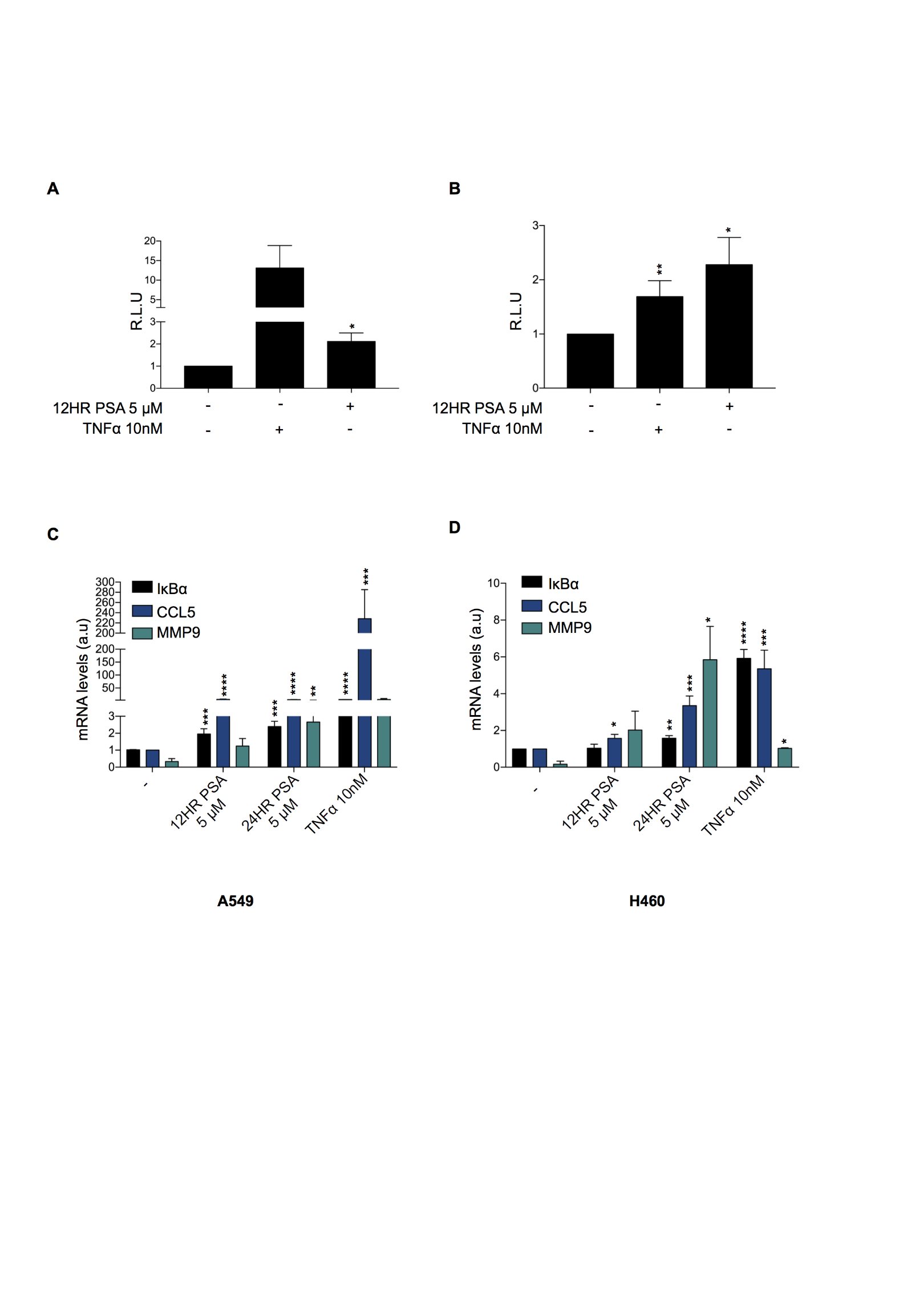


**Fig. S7. Psammaplin-A induces P65 transcriptional activity.**

**(A-B)** Luciferase assays of NF-κB activity in A549 and H460 cells treated with 5 μM Psammaplin-A or 10 nM TNFα for 12h. Data are shown as mean ± sem (n ≥ 3 independent experiments). P-values are from Student’s t-test. *P<0.05; **P<0.01 **(C-D)** Gene expression analysis by Real-time PCR of NF-κB target genes in A549 and H460 cells treated with 5μM Psammaplin-A or 10 nM TNFα for 12h. Data are shown as mean ± sem (n ≥ 3 independent experiments). P-values are from Student’s t-test. *P<0.05; **P<0.01; ***P<0.001; ****P<0.0001.

**
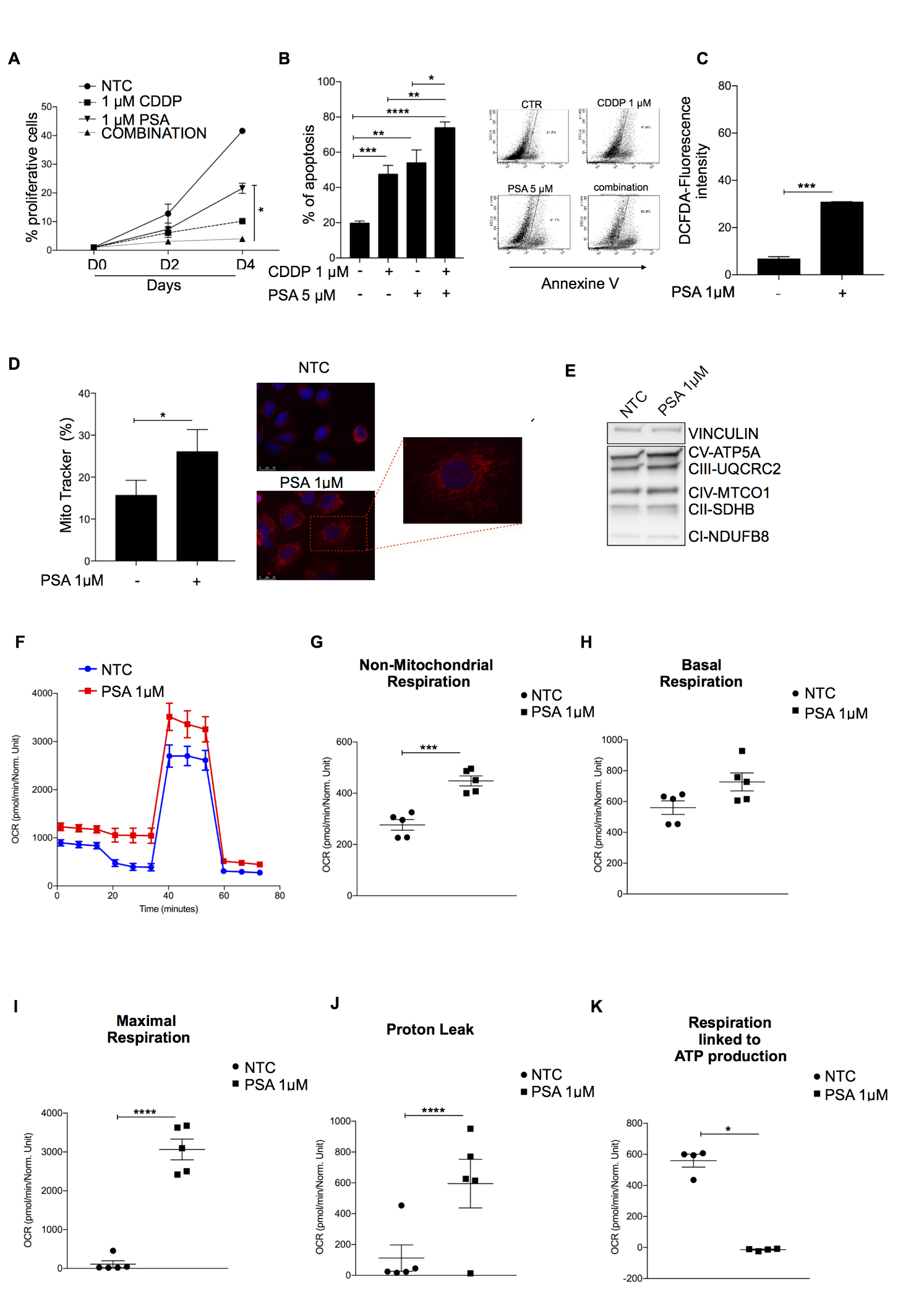
**

**Fig. S8. Psammaplin-A induces apoptosis via mitochondrial bioenergetics dysfunction**

**(A)** Growth curves of H460 cells treated with 1 µM cisplatin alone or in combination with 5 µM PSA for 96 h. Data are shown as mean ± sem (n ≥ 3 independent experiments). P-values are from Student’s t-test. *P<0.05. **(B)** Left panels Percentage of apoptotic H460 cells subjected to treatment with 1 µM cisplatin, 5 µM PSA or in combination with two drugs for 48 h, assessed by Annexin V Data are shown as mean ± sem (n ≥ 3 independent experiments). P-values are from Student’s t-test. *P<0.05; **P<0.01; ***P<0.001 ****P<0.0001. Right panel: Flow cytometry analysis of Annexine V in H460 cell line. The figure is a representative of three experiments with similar results. **(C)** Flow cytometry experiments measuring intracellular ROS levels in H460 cells treated with 1 µM PSA for 48 hr. Data are shown as mean ± sem (n ≥ 3 independent experiments). P-values are from Student’s t-test. ***P<0.001 **(D)** Left panel: quantification of mitochondria content. Data are shown as mean ± sem (n ≥ 3 independent experiments). P-values are from Student’s t-test. *P<0.05; Right panel: The representative images showed that PSA induced extensive mitochondrial fusion. **(E)** Representative Western blot showing five ETC proteins (ATP5A, ATP synthase, H+ transporting, mitochondrial F1 complex, α subunit; UQCR2, ubiquinol-cytochrome c reductase core protein II; SDHB, succinate dehydrogenase complex iron sulfur subunit B; COXII, mitochondrially encoded cytochrome c oxidase II; NDUFB8, NADH:ubiquinone oxidoreductase subunit B8) in H460 cells. I, II, III, IV, and V indicate ETC complexes. Vinculin is provided as a loading control. **(F)** Oxygen consumption rate (OCR, normalized using CyQUANT fluorescence, arbitrary units, AU). **(G)** Mean non-mitocondrial respiration, Mean basal respiration **(H)**, maximal respiration **(I),** proton leak **(J)** and respiration linked to ATP production **(K)** in cells treated with vehicle or PSA for 1 day (n ≥ 6). P-values are from Student’s t-test. ****P<0.0001; ***P<0.001; **P<0.01; *P<0.05

**
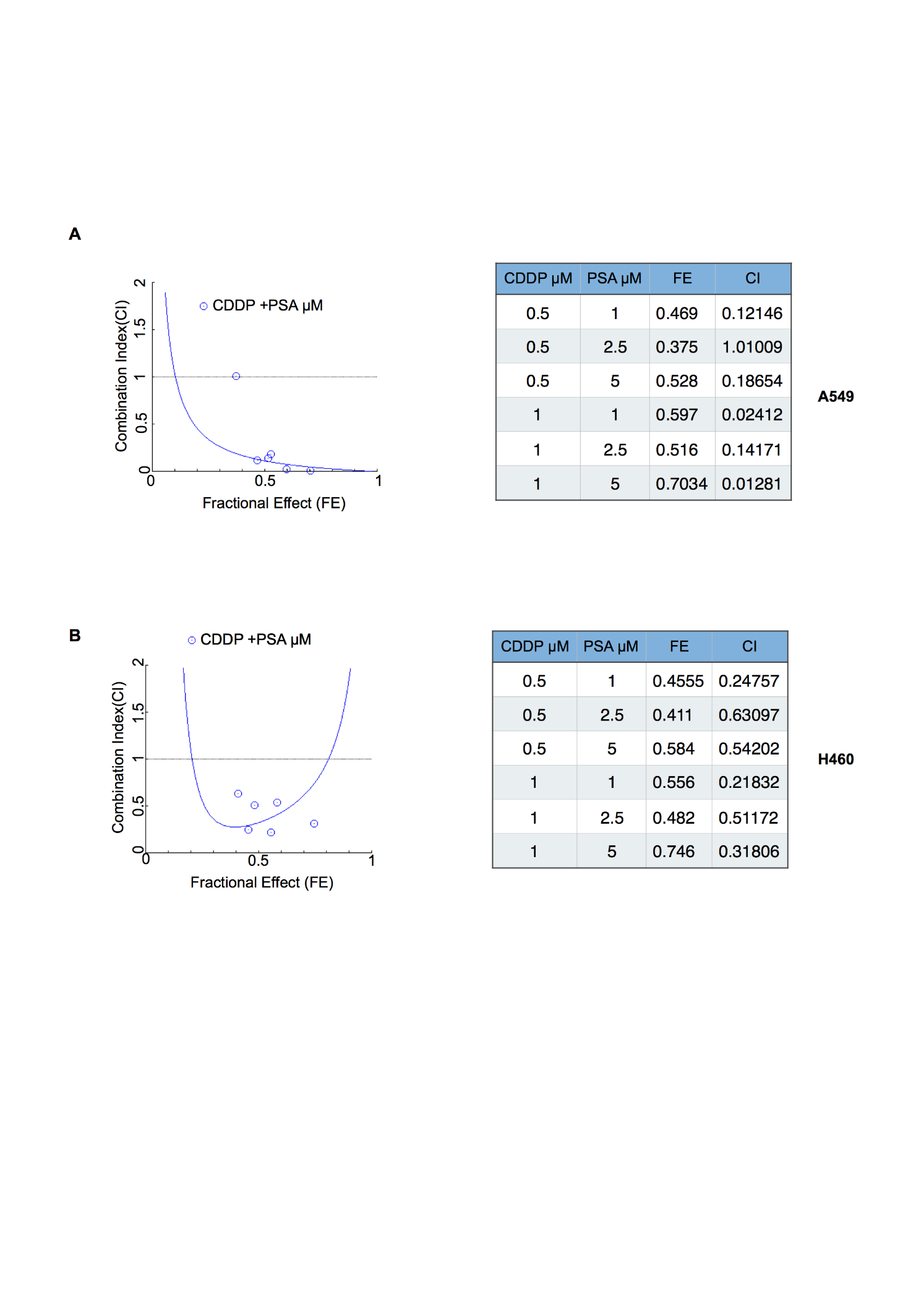
**

**Fig. S9. The combination of treatment with Cisplatin and Psammaplin-A exerts synergistic lethal activity against A549 and H460 cell lines.**

**(A-B)** A549 and H460 cell lines were treated with Cisplatin and Psammaplin-A at a constant ratio for 48 hours. The percent of apoptotic cells was determined by flow cytometry. Median dose effect and isobologram analyses were performed utilizing CompuSyn. CI values <1.0 indicates a synergistic interaction of the two agents in the combination.

**
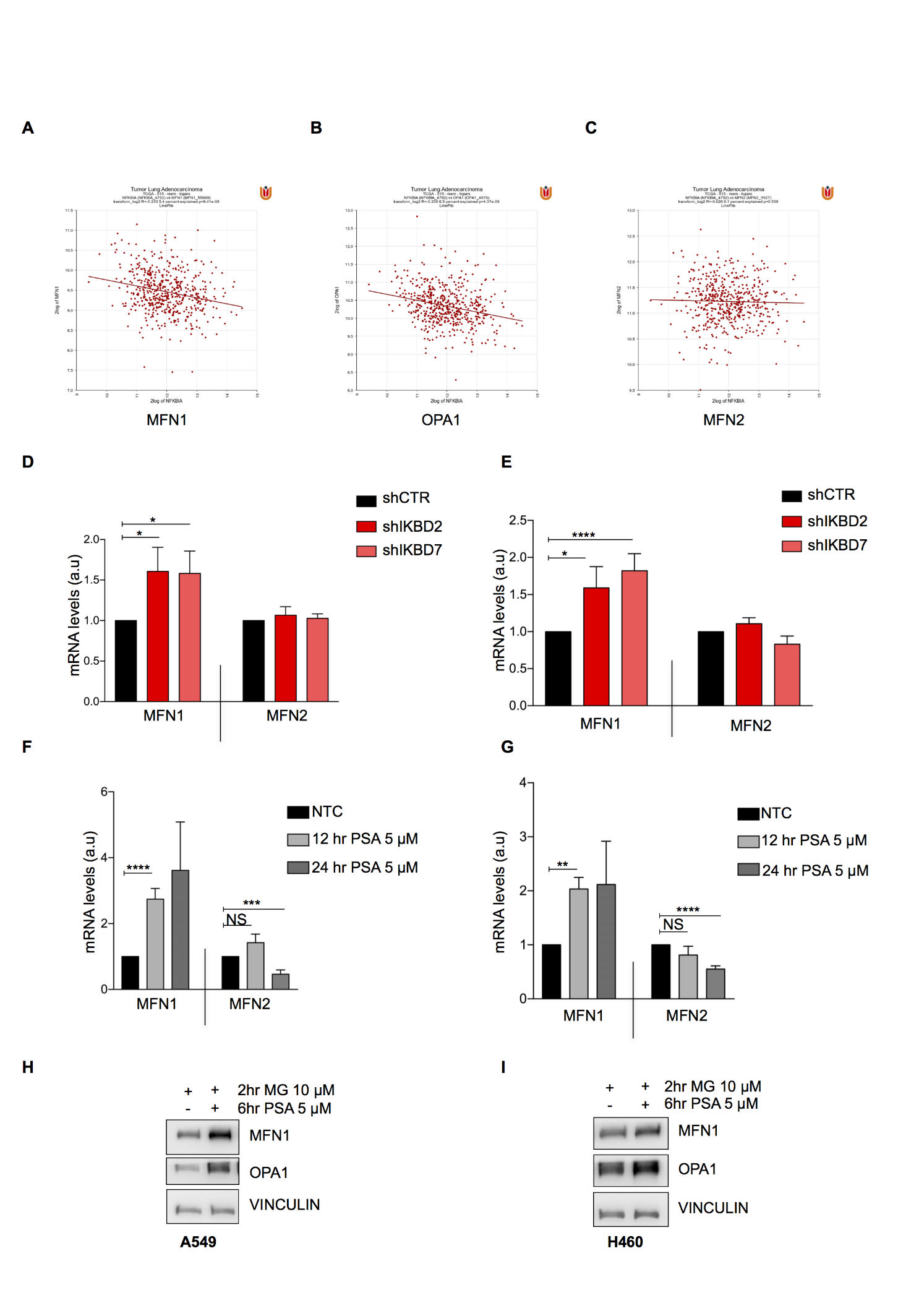
**

**Fig. S10. IκBα silencing or inhibition of the IκBα/p65 binding increase mitochondrial fusion markers.**

**(A-B-C)** Gene set enrichment analysis plot analyzed by the TCGA database of MFN1 (A), OPA1 (B) IκBα-inversely correlated and MFN2 (C). **(D-E)** Gene expression analysis by Real-time PCR of MFN1 and MFN2 in A549 and H460 cells infected with empty vector (shCTR) and two independent sh for IκBα (shIKBD2 and shIKBD7). Data are shown as mean ± sem (n ≥ 3 independent experiments). P-values are from Student’s t-test. *P<0.05; ***P<0.001. **(F-G)** mRNA levels of MFN1 and MFN2 in A549 and H460 cells treated with 5 μM PSA after 12 and 24 h. Data are shown as mean ± sem (n ≥ 3 independent experiments). P-values are from Student’s t-test. **P<0.01; ***P<0.001; ****P<0.0001. **(H-I)** Western Blot analysis of MFN1 and OPA1 in A549 and H460 cells treated with 10μM MG132 for 2 h and 5 μM PSA as indicated.


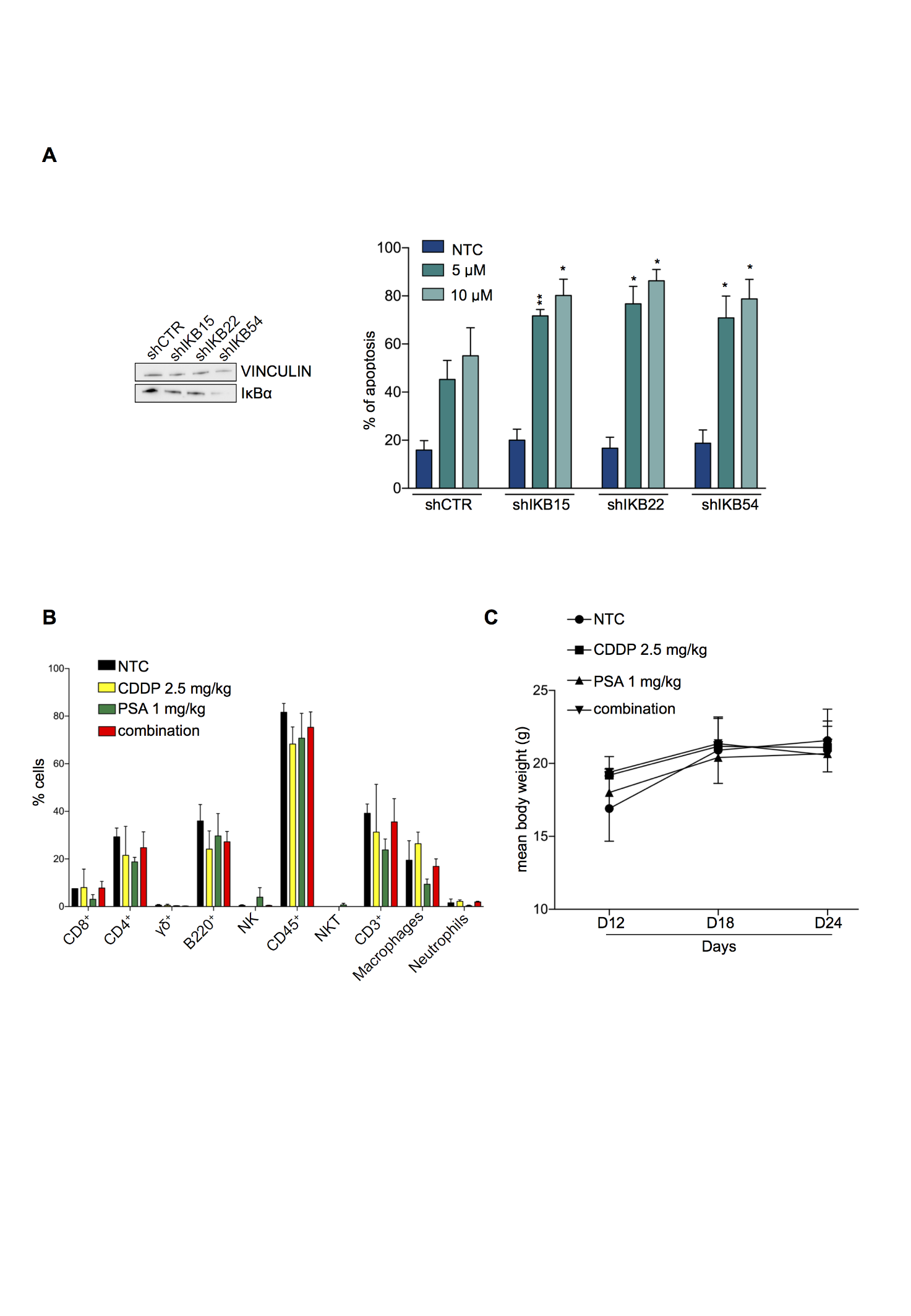


**Fig. S11. Analyses for PSA toxicity.**

**(A)** Western blot analysis of LLC cells infected with empty vector (shCTR) and three independent sh for IκBα (shIKB15, shIKB22 and shIKB54) immunostained IκBα and VINCULIN as loading control. Percentage of apoptotic LLC cells treated with 5µM and 10µM Cisplatin for 48 h, assessed by Annexin V. Data are shown as mean ± sem (n ≥ 3 independent experiments). P-values are from Student’s t-test. *P<0.05; **P<0.01 **(B)** Percentage of immune cells detected in blood of mice, 24 days after injection of LLC cells, treated with 2.5 mg/kg cisplatin, 1 mg/kg, alone or in combination. Data shows average ± Sem. **(C)** Graph representing the body weight of C57BL/6 mice treated with 2.5 mg/kg cisplatin, 1 mg/kg, alone or in combination. Data shows average ± Sem.

**Table S1. List of cancers type of TCGA cohorts**

**
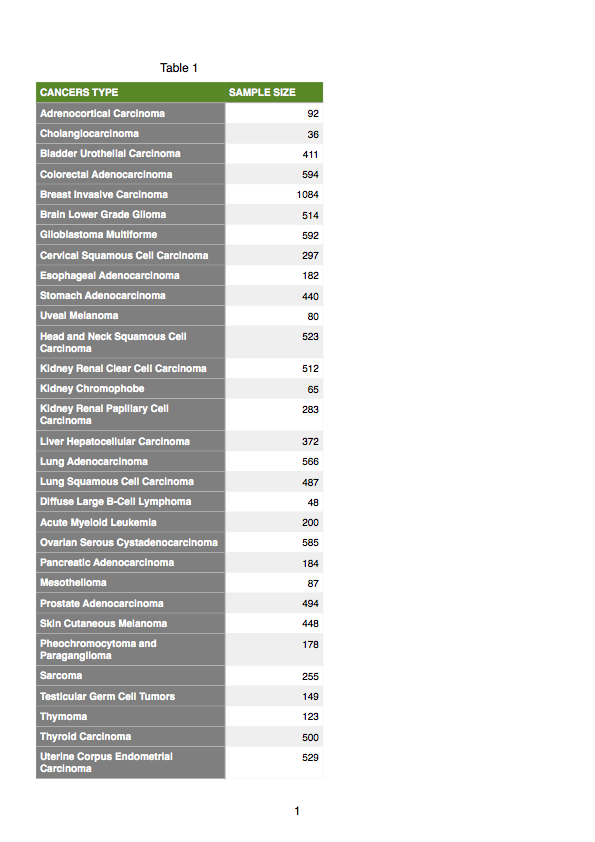
**

**Table S2. Perceptual of NFKBIA copy number variations in TCGA cohorts.**

**
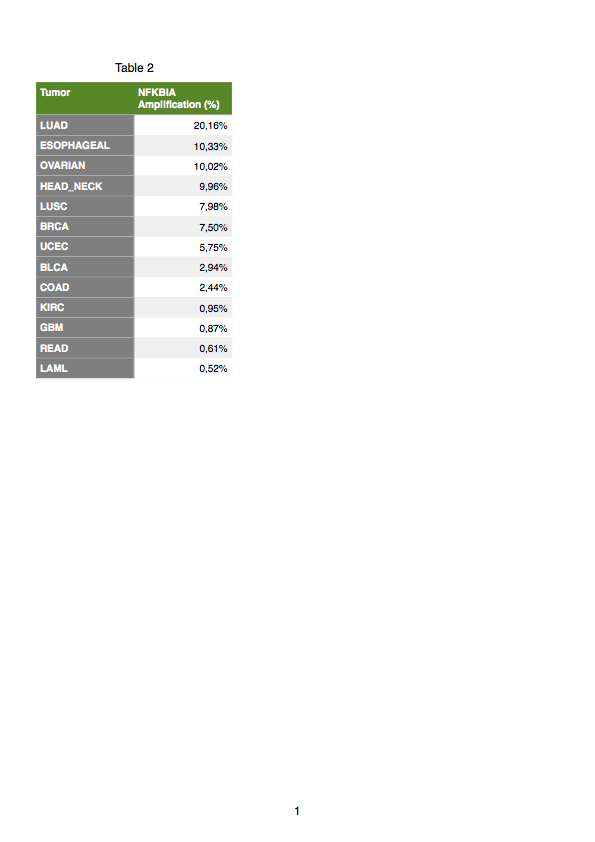
**

**
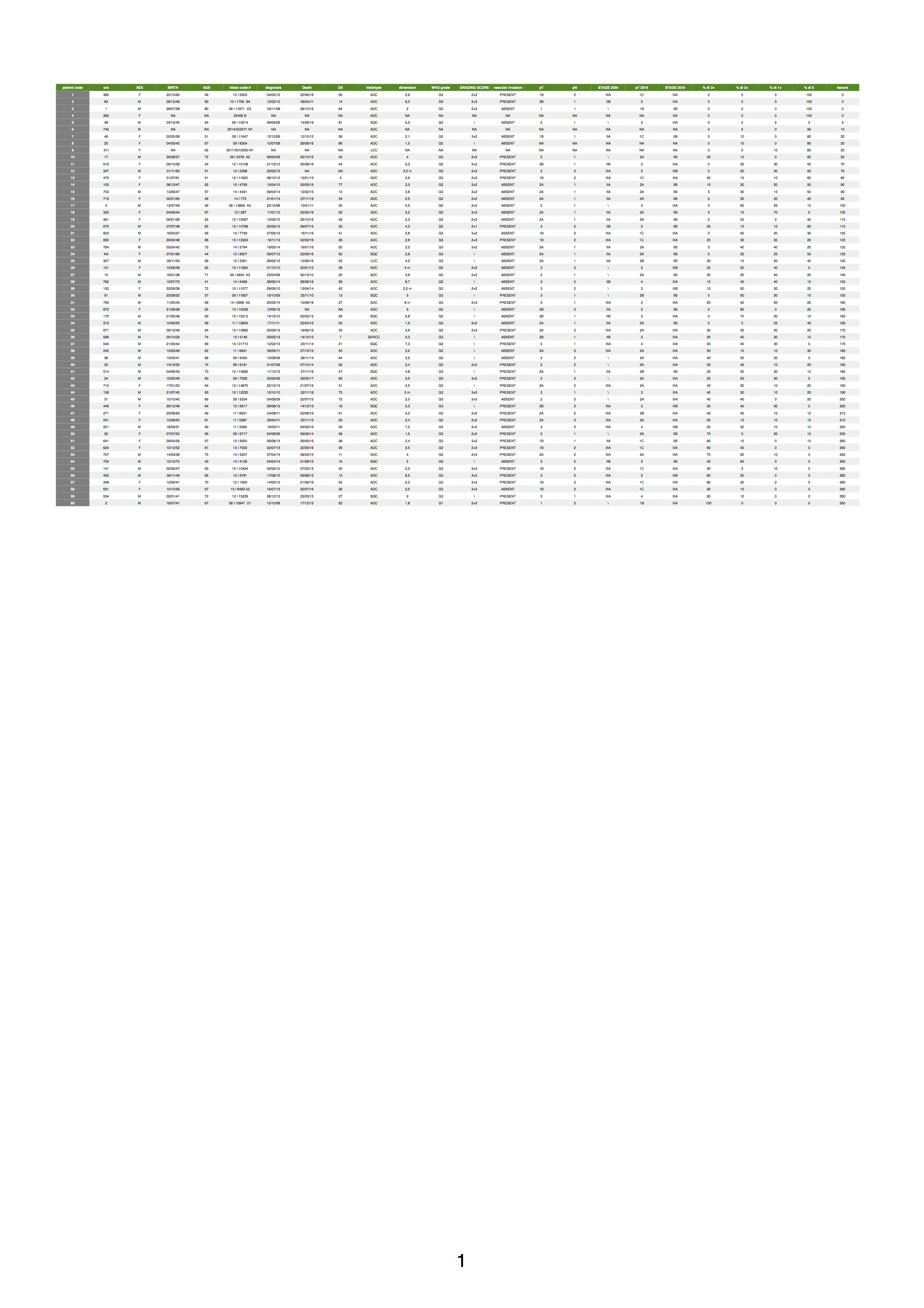
Table S3. Description of the lung patient’s information and relative HS score distribution**
